# Low soil moisture predisposes field-grown chickpea plants to dry root rot disease: evidence from simulation modeling and correlation analysis

**DOI:** 10.1038/s41598-021-85928-6

**Published:** 2021-03-22

**Authors:** Ranjita Sinha, Vadivelmurugan Irulappan, Basavanagouda S. Patil, Puli Chandra Obul Reddy, Venkategowda Ramegowda, Basavaiah Mohan-Raju, Krishnappa Rangappa, Harvinder Kumar Singh, Sharad Bhartiya, Muthappa Senthil-Kumar

**Affiliations:** 1 National Institute of Plant Genome Research, Aruna Asaf Ali Marg, P.O. Box No. 10531, New Delhi, 110067 India; 2 ICAR-IARI-Regional Research Center, P. B. Road, Dharwad, 580001 India; 3 Department of Botany, Yogi Vemana University, Kadapa, Andhra Pradesh 516005 India; 4 Department of Crop Physiology, University of Agricultural Sciences, GKVK, Bangalore, 560 065 India; 5 Division of Crop Production, ICAR Research Complex for North Eastern Hill Region, Umroi Road, Umiam, 793103 India; 6 Department of Plant Pathology, Indira Gandhi Krishi Vishwavidyalaya, Raipur, 492012 India; 7 Department of Chemical Engineering, Indian Institute of Technology Bombay, Powai, Mumbai 400076 India

**Keywords:** Biological techniques, Plant sciences

## Abstract

*Rhizoctonia bataticola* causes dry root rot (DRR), a devastating disease in chickpea (*Cicer arietinum*). DRR incidence increases under water deficit stress and high temperature. However, the roles of other edaphic and environmental factors remain unclear. Here, we performed an artificial neural network (ANN)-based prediction of DRR incidence considering DRR incidence data from previous reports and weather factors. ANN-based prediction using the backpropagation algorithm showed that the combination of total rainfall from November to January of the chickpea-growing season and average maximum temperature of the months October and November is crucial in determining DRR occurrence in chickpea fields. The prediction accuracy of DRR incidence was 84.6% with the validation dataset. Field trials at seven different locations in India with combination of low soil moisture and pathogen stress treatments confirmed the impact of low soil moisture on DRR incidence under different agroclimatic zones and helped in determining the correlation of soil factors with DRR incidence. Soil phosphorus, potassium, organic carbon, and clay content were positively correlated with DRR incidence, while soil silt content was negatively correlated. Our results establish the role of edaphic and other weather factors in chickpea DRR disease incidence. Our ANN-based model will allow the location-specific prediction of DRR incidence, enabling efficient decision-making in chickpea cultivation to minimize yield loss.

## Introduction

Field crops are routinely exposed to multiple abiotic and biotic stresses. Often, two or more stresses are influenced by each other, and the environmental factors^1–5^. Dry root rot (DRR), one of the major diseases of chickpea (*Cicer arietinum*), is caused by the necrotrophc fungus *Rhizoctonia bataticola*. DRR is reported to cause up to 100% yield loss in chickpea under conditions favouring *R. bataticola* growth^6^. DRR in chickpea has been reported to increase under low soil moisture and high temperature conditions^5,7–11^. DRR disease incidence is also reported to be influenced by soil pH, with the highest DRR incidence occurring at pH 5.0^10^. Similarly, soil type has an impact on disease incidence with clayey and sandy soil characteristics supporting DRR occurrence^10,12^. Nevertheless, comprehensive information is not available on the possible role of major environmental and edaphic factors in DRR incidence and hence making it difficult to assess and predict DRR incidence in field-grown chickpea plants. Altering sowing time/location, application of soil amendments and irrigation schedule that otherwise predispose plants to DRR disease development can be used to manage the disease to an extent. Therefore, it is imperative to decipher how environmental and edaphic factors are influencing DRR disease incidence and it is essential to formulate or model this finding for effective disease incidence prediction.

Artificial neural network (ANN) is a very efficient machine learning tool for building forecasting models of complex and nonlinear systems with a large number of predictor variables^13^. The network modelling in ANN is data driven, and with suitable input (predictor) and output (response) data, it can efficiently establish relationships with reliable predictability. Machine learning methods like multiple linear regression (MLR), ANN methods like backpropagation neural network (BPNN), generalized regression neural network (GRNN), recurrent neural networks (RNNs), and long short-term memory networks (LSTMs), and support vector machine (SVM) have been have been successfully applied to establish plant–pathogen–environment interactions and efficient disease predictions in crop plants such as rice, wheat, and maize^14–19^. ANN has also been used to establish relationships between crop yield and edaphic and environmental factors in major crops like wheat, barley, soybean, sorghum, and maize^20–24^.

In this study, we used an ANN-based simulation modeling approach to predict the possible contribution of environmental and edhaphic factors to DRR incidence using public resources. These predictions are validated through multi-location field experiments. Correlation between different factors and DRR disease incidence are providing comprehensive information that might prove valuable in controlling DRR disease during chickpea cultivation.

## Results

### Mining and curating DRR disease incidence and weather data from publicly available resources

To understand the factors influencing DRR incidence, we collected research articles and theses published during 2009–2018 focusing on DRR incidence in field-grown chickpea at multiple locations across India (Supplemenary Table S1). These studies included several geographic locations, percent disease incidences, soil types, and genotypes. The datasets included 11 genotypes, regions with black soil, red soil, and clay soil, irrigated and rainfed fields. Data from major soil types (black and red soil) and genotypes (JG11 and Annigeri 1, i.e., A1) were selected for soil and geneotype specific analysis (Supplementary File S1) as these two genotypes are majorly cultivated in southern region of the India contributing to a large percentage of total world production. Also, DRR disease information is majorly available for these two genotypes and black and red soil type.

The weather data obtained from the India meteorological department were analysed for the rabi season (October to February), for the year when field experiments were conducted (e.g., 2009–2010). The total monthly rainfall and average monthly temperature were calculated for October to February months (Supplementary File S1) as chickpea are shown in October and harvested in month of March in the area considered mentioned in this study. Further, total rainfall in two months and similarly three months and average minimum and maximum temperatures for the two months and three months starting (e.g. October + November, November + December etc.) were calculated (Supplementary file S1) to closely check which period rainfall is mostly impacting the DRR incidence. First, correlation analyses were performed with publicly available DRR disease incidence data to study the relation between DRR disease incidence and weather components rainfall and temperature/impact of weather factors on DRR disease incidence. Further, ANN training was used to establish relation weather/edaphic factors and DRR incidence under field condition.

### DRR disease incidence was negatively correlated with rainfall and positively correlated with atmospheric temperature

**Figure 1:**
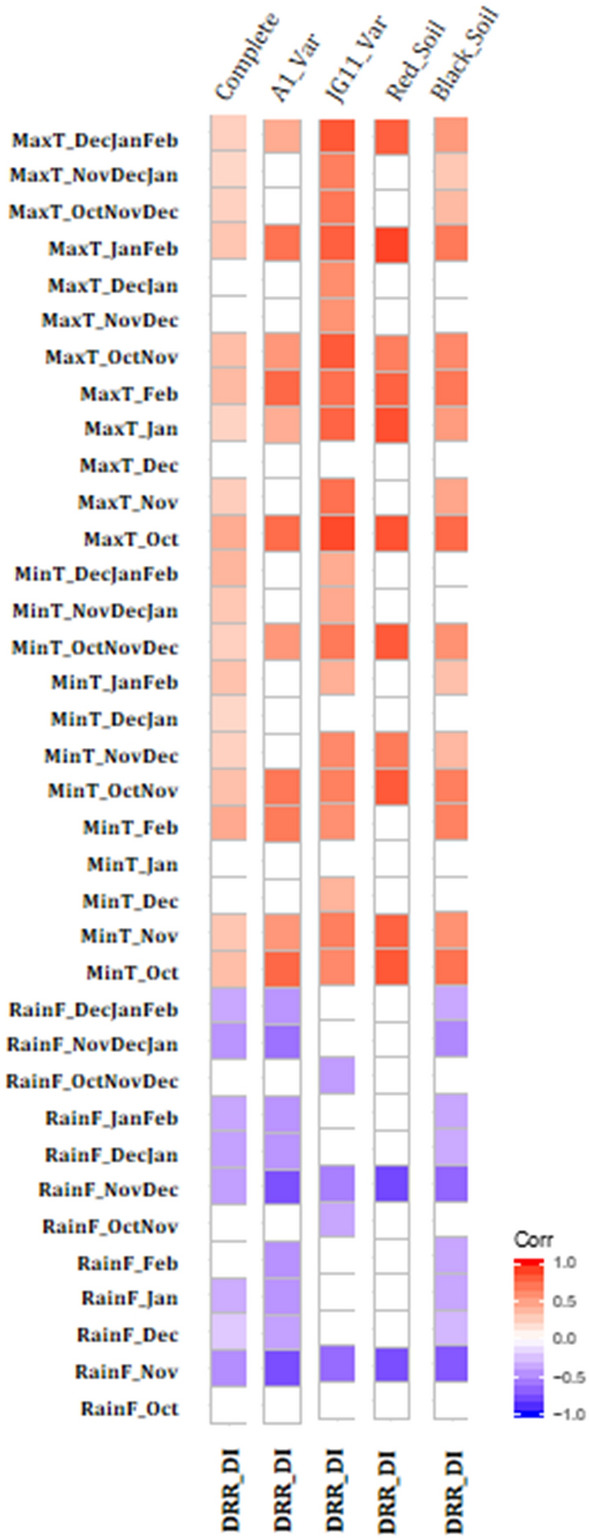
Correlation analyses between DRR disease incidence and weather parameters. Correlation analyses was performed using publicly available DRR disease incidence data to study the relation between DRR disease incidence and weather components; rainfall and temperature. DRR disease incidence data was gathered from published research articles and theses (Supplementary file S1, Supplementary Table S1). Weather data for the respective locations were acquired from Indian Meteorological Department. Weather data for the field trial season mentioned in the source was acquired, and the monthly average for the months from October to February for maximum and minimum temperature was calculated. Total monthly rainfall and total rainfall for the two and three consecutive months were also calculated. A correlation was performed using Pearson’s correlation method for all the possible weather factors and DRR Disease incidence (a). Correlation analyses was performed for complete DRR data set, and data set for the A1 genotype, JG11 genotype, red soil type and black soil type. Red boxes represents significant positive correlation, and blue boxes represent significant negative correlation, empty boxes represent no significant correlation. Correlation with p < 0.05 was taken as statistically significant. A negative correlation exists between DRR disease incidence and rainfall, while a positive correlation exists between disease incidence and minimum and maximum temperature. RainF = rainfall, MinT = minimum temperature, MaxT = Maximum temperature, DRR DI = DRR disease incidence, Oct = October, Nov = November, Dec = December, Jan = January, Feb = February, Mar = March.

Pearson’s correlation was performed between DRR disease incidence, monthly, two-months, three-months total rainfall, an average of monthly minimum and maximum temperature. A correlation matrix was developed in R. Correlation analyses showed a negative correlation between DRR disease incidence and rainfall. A correlation of − 0.49 was observed between DRR disease incidence and the total rainfall during November, and a correlation of − 0.45 was observed between DRR disease incidence and total rainfall from November to January (Fig. 1). A positive correlation was observed between DRR disease incidence and minimum and maximum atmospheric temperature. The correlation between DRR disease incidence and the average minimum temperature of February was 0.43. Similarly, the correlation values between DRR disease incidence and average maximum temperature of October and February were 0.42 and 0.36, respectively (Fig. 1, Supplementary Fig. S2a, Supplementary File S1). Since the total data used in the above correlation analyses are from varied sources, the variation caused by genotype and soil type can affect the correlation between DRR and weather factors. Therefore, we also analysed the genotype-wise and soil category-wise correlation of DRR disease incidence with rainfall and temperature. For both A1 and JG11 genotypes, DRR incidence was significantly correlated with the total rainfall of November (− 0.75 and − 0.64, respectively) and November to December (− 0.75 and − 0.54, respectively). The DRR incidence in genotype A1 was positively correlated with the average minimum and maximum temperature for October and February (0.74, 0.66, 0.72, 0.74, Fig. 1, Supplementary Fig. S2b, Supplementary File S1). For genotype JG11, a positive correlation was observed between DRR disease incidence and the average minimum temperature of October (0.60) and November (0.64). Similarly, DRR incidence showed a significant positive correlation with the average maximum temperature for October (0.85), November (0.71), January (0.75), and February (0.71) (Fig. 1, Supplementary Fig. S2c).

For both black and red soil types, DRR disease incidence was highly correlated with the total rainfall in November (− 0.71, − 0.75) and November to December (− 0.66, − 0.78). The average minimum temperature of October and November had a good correlation with DRR disease incidence for black soil (0.69, 0.65). In contrast, DRR incidence was highly correlated with the average minimum temperature for October (0.81) and November (0.76) for red soil (Fig. 1, Supplementary Fig. S2d and S2e). For black soil, DRR disease incidence was positively correlated with the average maximum temperature in October (0.73) and February (0.68). Moreover, for red soil, the average maximum temperatures of January–February, and December–February were highly correlated with DRR disease incidence (0.88, 0.78) (Fig. 1, Supplementary Fig. S2d and S2e).

The negative correlation between DRR disease incidence and rainfall in the case of both the genotypes and both the soil types indicates that rainfall negatively influences DRR disease incidence irrespective of genotype or soil type. The positive correlation of the average maximum temperature of October with DRR disease incidence could be because of the influence of increased temperature on *R. bataticola* infectivity or its influence on soil moisture status.

### Training of ANN and validation of the output

**Figure 2:**
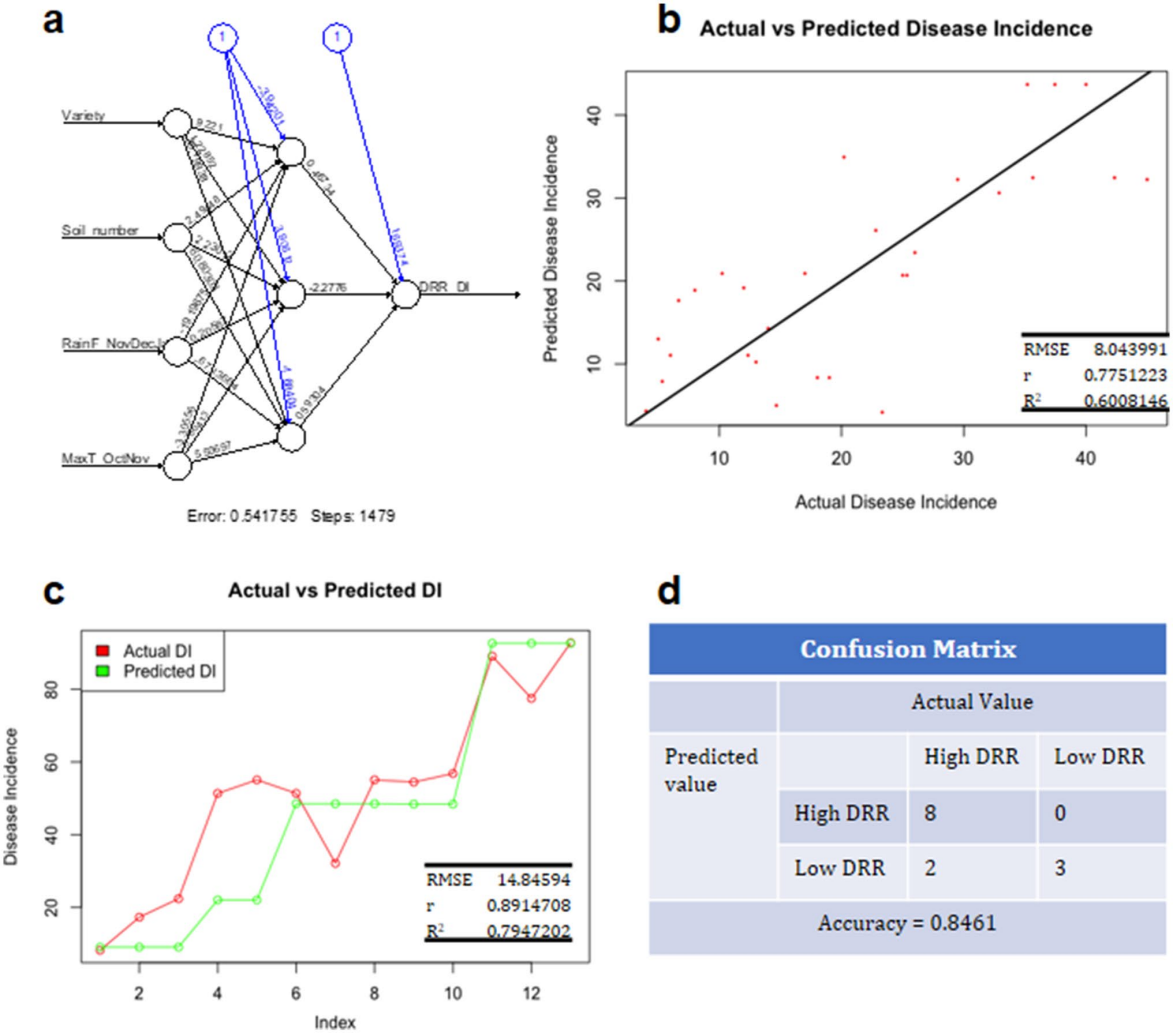
Training, testing, and validation of neural network. Neural network was trained using total rainfall from November to January (RainF_NovDecJan), an average of maximum temperature for the October and November months (MaxT_JanFeb) along with information about variety (Variety) and soil type (Soil_number) as input, and DRR disease incidence data (Supplementary File S1) as output. The entire dataset was divided into 70% training set and 30% testing set. Neural network (NN) was trained in R using neuralnet algorithm with backpropagation method. The best fit in NN training was obtained with one hidden layer having three nodes. Network topology for the training and linear regression between actual and predicted for the training set are shown in (**a**) and (**b**), respectively. Numbers in connecting lines from input layer to hidden layer and from hidden layer to output layer represent weights used in the model and number connecting blue circles are biases. Linear activation function was used for the network training. Validation of the trained neural network was performed with DRR disease incidence from the current (2019–2020) field experiment study and with DRR incidence data of location-2 from Sinha et al. (2019) research article. Validation data set included DRR disease incidence data from severe combined stress treatment plots. Input and output data used for validation is in Supplementary File S1. A comparison between actual (red) and predicted DRR disease incidence (green) is shown as line graph (**d**). RMSE, r and R^2^ is showing root mean square error, correlation coefficient and coefficient of determination, respectively. DRR incidence was categorized into High DRR (disease incidence > 30%) and Low DRR (disease incidence < 30%) and confusion matrix was created with actual and predicted DRR incidence for the validation set. The prediction accuracy was calculated.

The pipeline for ANN-based simulation is outlined in Supplementary Fig. S1. ANN training was performed with chickpea genotype (Variety), soil type (Soil_number), and the data of weather factors as input variables, and DRR disease incidence as the output (single output). The neuralnet algorithm with backpropagation algorithm with a linear activation was used for the training in R. The data set was scaled using min–max normalization method to scale the data between 0–1 before ANN training. Different hidden layers were tried during training to select the most suitable hidden layer for the best fit. The entire dataset (of 96 datapoint) for the neural network training was divided into 70% training set (67 data points) and 30% testing set (29 data points). Prediction accuracy of the neural network was tested with calculated root mean square error (RMSE), coefficient of regression (r), and coefficient of determination (R^2^) between actual DRR disease incidence of the testing set and predicted disease incidence for the testing set. Various combinations of weather inputs were used for ANN training before selecting the best-fit model (Supplementary Table S2a). Also, K-fold cross validation was used for the selection of best fit model (Supplementary Table S2b). When only one weather factor, i.e., either rainfall or temperature, was included with soil type and genotype as input for ANN training, the coefficient of correlation between the predicted and actual DRR incidence ranged from 0.35 to 0.72 (Supplementary Table S2a). Furthermore, combinations of weather input parameters–rainfall and maximum temperature–were used with genotype and soil type for ANN training. Out of 144 combinations of four inputs, the top 32 combinations with the highest r values were selected and rechecked by k-fold cross-validation (k = 10). The network trained with the input combination of Rainfall_NovDecJan, MaxT_OctNov, genotype, and soil type was found to have the highest r, RMSE, and R^2^ between actual and predicted values (Supplementary Table S2b). Network topographs for the training and linear regression between actual and predicted values for the training set are shown in Fig. 2a, b, respectively. The best trained neural network with one hidden layer and three nodes (Fig. 2a) with linear activation function showed an RMSE of 8.04, r of 0.775, and R^2^ of 0.600 between actual and predicted DRR incidence for the testing set (Fig. 2b). The network was validated using DRR disease incidence data from location-4 of a current year trial and a dataset from Sinha et al. (2019) (Supplementary File S1). The RMSE, r, and R^2^ between the actual and predicted DRR disease incidence from the validation dataset were 14.84, 0.89, and 0.79, respectively. Figure 2c shows line graphs for actual and predicted DRR disease incidence. Further, the prediction accuracy was checked by using a confusion matrix, and the accuracy was found to be 0.84 (Fig. 2d).

### Impact of edaphic factors on DRR disease incidence: evidence from field experiments

**Figure 3:**
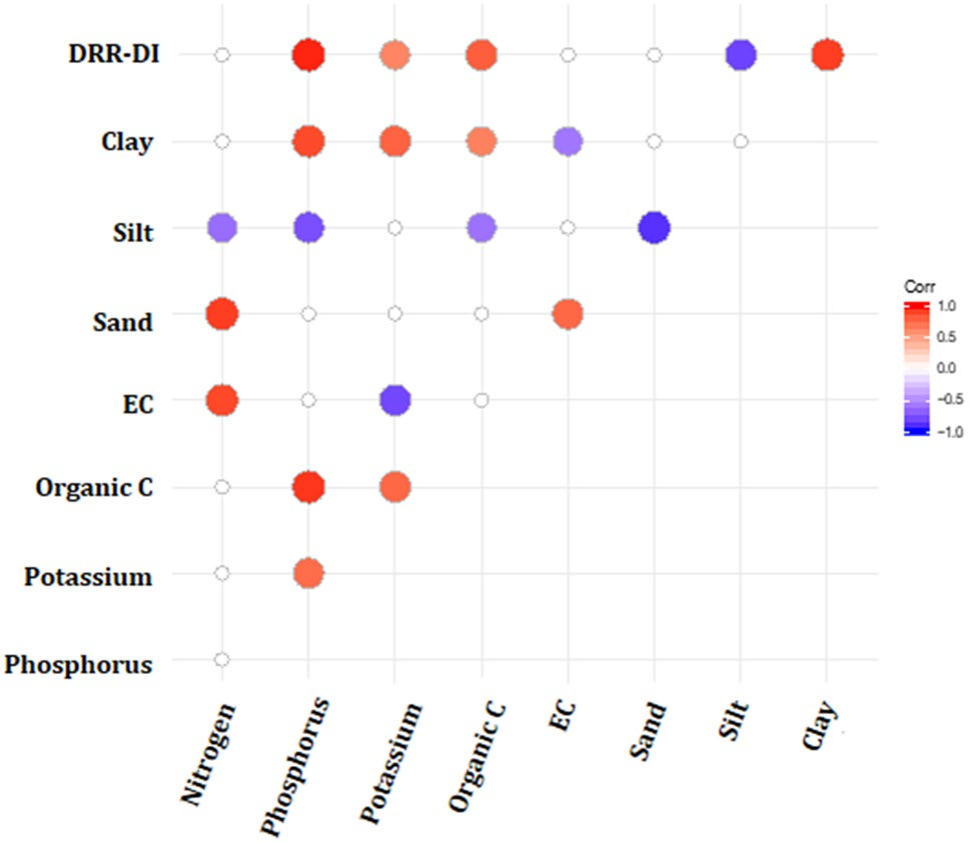
Correlation between DRR disease incidence and various edaphic factors. DRR disease incidence data from the field trial year 2018–19 and 2019–20 were analyzed for correlation (Pearson) with the edaphic factors for the respective trials. Red circles in correlation matrix represent significant positive correlation, and blue circles represent significant negative correlation, empty circles represent no significant correlation. Correlation with p < 0.05 was taken as statistically significant. Data used for the analyses along with correlation matrix with *p-*value is in Supplementary Table S3. DRR-DI = DRR disease incidence, EC = electrical conductivity of soil, organic C = organic carbon of soil.

To assess the relation between low soil moisture/edaphic factors and DRR disease, field experiment with low to normal irrigation were performed at seven different geographical locations in India. Details of the location are in Supplementary Fig. S3. The treatments in the field experiemnts were low moisture stress (LSM), pathogen stress (PS), combined stress (CS), along with control in four replicates in RCB design (Supplementary Fig. S4). Soil composition and properties (Supplementary Table S3a) were assessed and disease incidence was calculated for all treatments. Majorly, content for nitrogen (N), Phosphorus (P), Potassium (K), organic carbon (OC), along with electrical conductivity, sand, silt and clay content have been measured. Location -2 and Location-3 had soil with high sand content whereas Location-1 and Location-4 had higher silt content. Location-2 and Location-4 had higher P, K and OC content compared to other locations (Supplementary Table S3a). DRR disease incidence data from locations 1–4 from the field experiment of the year 2019–2020 were analysed for Pearson’s correlation with the edaphic factor data for the respective trials (Supplementary File S1). To eliminate the effect of the soil moisture component, which has been found to be a major influence, and to focus on other edaphic factors, DRR incidence data were considered from PS treatment plots (Supplementary File S1, Supplementary Table S3a). With a correlation coefficient of 0.96, phosphorus (P) showed a significant positive correlation (p-value 3.62E−07) with DRR disease incidence (Fig. 3, Supplementary Table S3b, S3c). Similarly, potassium (K) and total organic carbon (OC) had a positive correlation, with correlation coefficients of 0.62 (p-value 0.022) and 0.78 (p-value 0.0013), respectively (Fig. 3, Supplementary Table S3b, S3c). Clay content of the soil was positively correlated with DRR incidence (correlation coefficient, 0.89, p-value 3.47E-05), while silt content showed a significant negative correlation (correlation coefficient, − 0.81, p-value 0.00077) (Fig. 3, Supplementary Table S3b, S3c). Although electrical conductivity (EC) did not show a direct significant correlation, it showed a negative correlation with clay content, implying a negative impact on DRR disease incidence (Fig. 3, Supplementary Table S3b, S3c).

### DRR disease incidence increased under low soil moisture stress: evidence from field experiments

**Figure 4:**
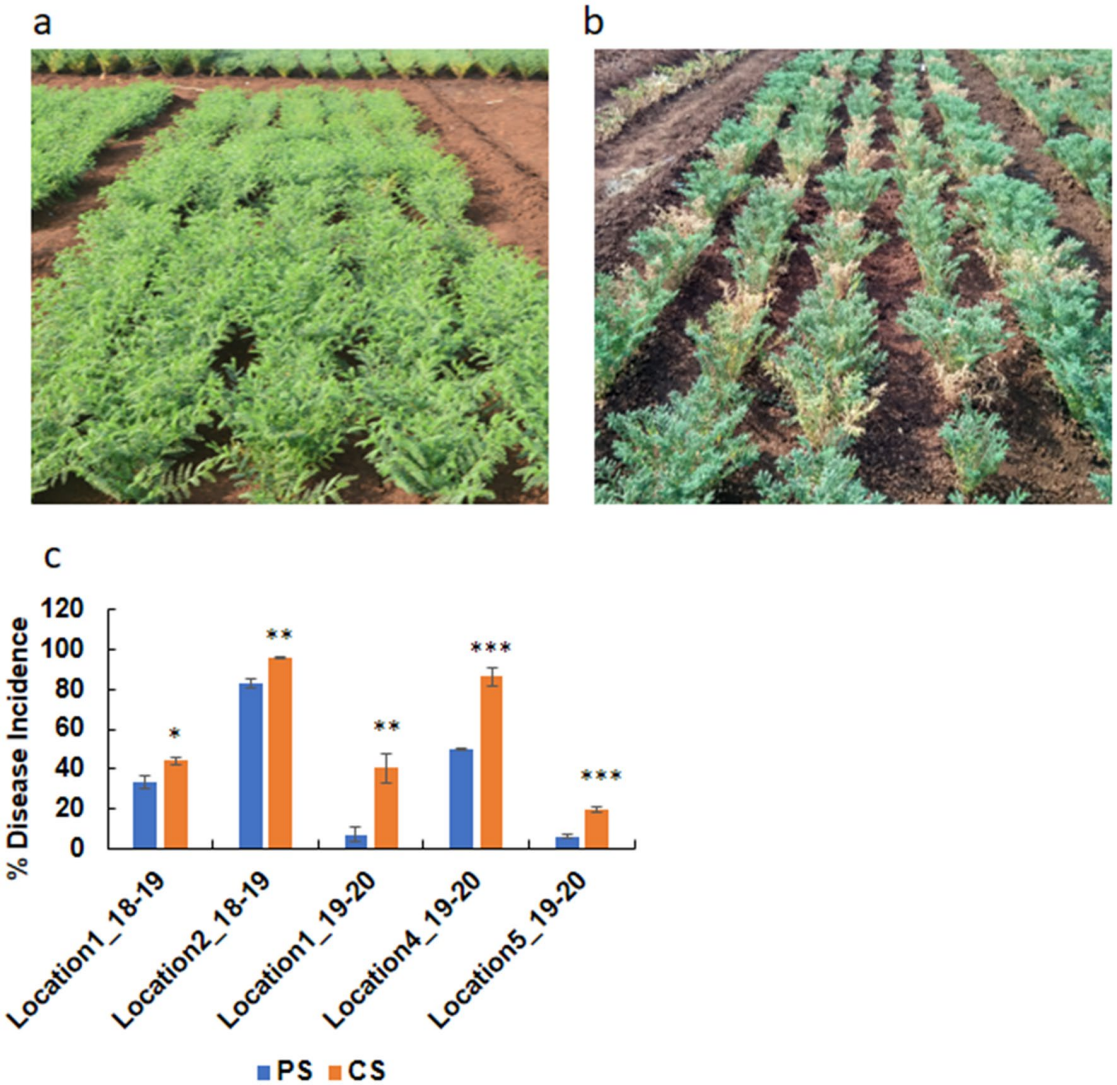
Dry root rot disease incidence under pathogen, and combined stress treatments in field trials. Field experiment was conducted at three different geographical locations during 2018–2019 and seven different geographical locations during 2019–2020 in India (Supplementary Fig. S5) with control, PS, LSM, and CS (Supplementary Fig. S6). The experiment was conducted with four replicates in RCB design. The results from PS and CS treatments were analysed to understand the impact of low soil moisture on DRR incidence. The field trial layout (representative) for PS treatment and CS treatment are shown in figure (**a**) and (**b**), respectively. Percent DRR disease incidence calculated during the field trial period are shown for five field trial location in the graph (**c**). All the DRR disease incidence is an average of three to four RCB replicates with Standard error of mean. Significance difference between means were analysed by *Student’s t test*. *p < 0.05, **p < 0.005, ***p < 000.5. PS, pathogen stress; CS, combined low soil moisture and pathogen stress.

**Figure 5:**
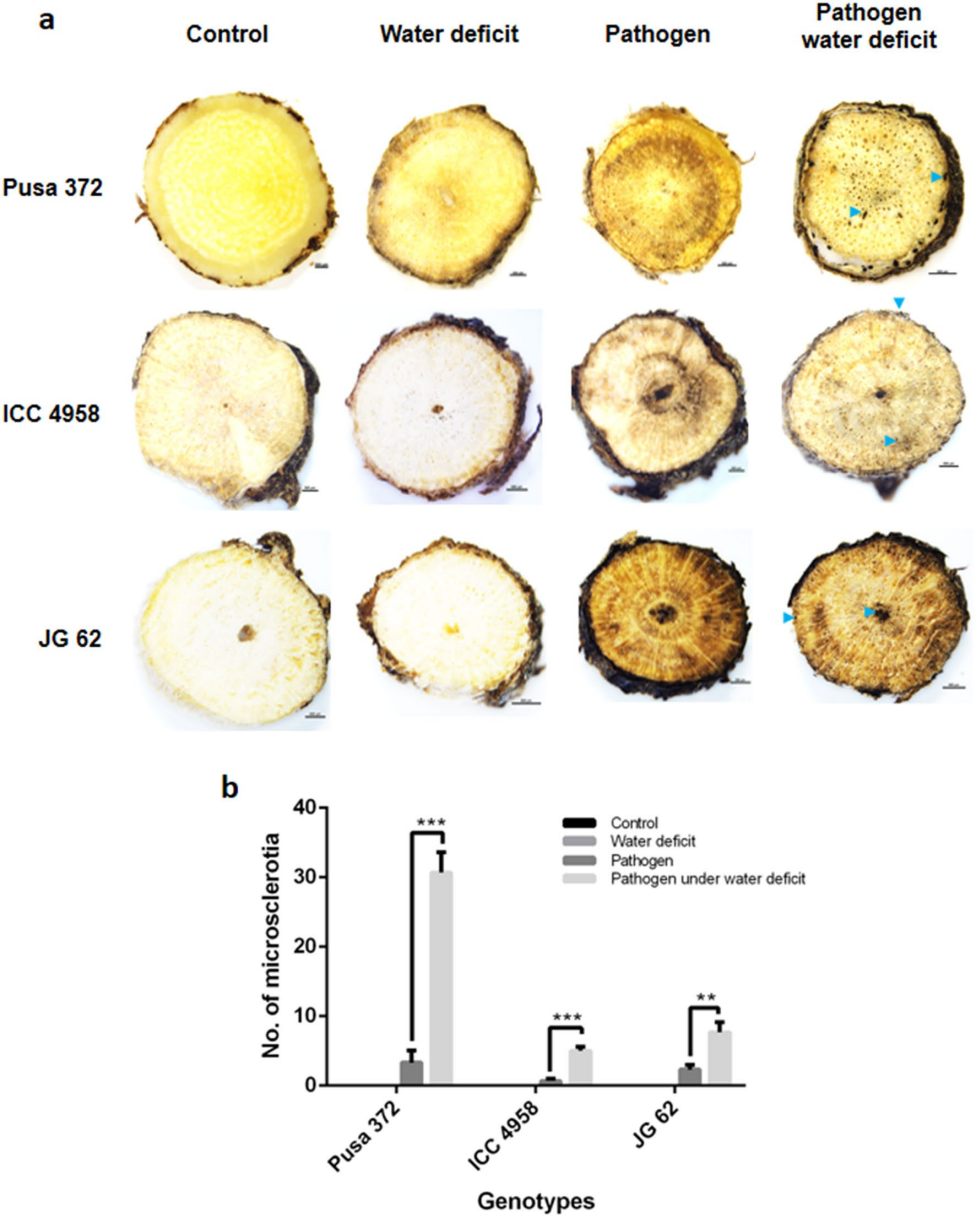
Low soil moisture aggravates the DRR incidence in various chickpea genotypes. Chickpea genotypes PUSA 372, ICC 4958 and JG 62 were used to explore the impact of soil water deficit stress on DRR disease incidence. The experiment was conducted during the rabi season (October to March, 2019–2020). Plants were imposed with LSM, PS, and CS treatments and along with control. Plants were uprooted at the time of maturity and examined for the presence of microsclerotia in the root. Handmade root sections were made at 2 cm from the point of seed attachment. Microsclerotia present on the root was counted. Images (**a**) show the transverse section of three genotypes in all the treatments and control. Graph (**b**) shows the number of microsclerotia in the root sections. Images were captured under the 0.5X objective lens of the research stereo microscope SMZ25. Scale bar is 500 µm. N = 3. Significance difference was determined using one-way ANOVA (p < 0.0001) and the asterisk represents the significance. Blue arrows show the microsclerotia. LSM, low soil moisture; PS, pathogen stress; CS, combined low soil moisture and pathogen stress.

The soil moisture content in all the field trial locations was significantly lower in LSM and CS treatments compared to control and PS treatments. The soil moisture content in LSM and CS was 5–10%, whereas in the control and PS, it was around 15–25% (Supplementary Fig. S7). The differential irrigation regimen also affected chickpea plant growth in the treatment plots (Supplementary Fig. S5). The influence of weather factors, namely average temperature, average humidity, and total rainfall in field trial locations across years, was studied (Supplementary Fig. S8). The results showed that soil moisture was the significantly influencing parameter of DRR disease incidence compared to other parameters. The DRR disease incidence during the trial year 2018–2019 in location-1 significantly increased to 44.13% under CS treatment compared to 33.56% disease incidence in the PS treatment (Fig. 4). Similarly, disease incidence increased from 83.07% in the PS treatment to 95.98% in the CS treatment in location-2 during the 2018–2019 trial. Field trials conducted during 2019–2020 also showed increased DRR disease incidence in CS plots compared to PS treatment in locations 1, 4, and 5 (Fig. 4). The disease severity index also increased under CS compared to the PS treatment (Supplementary Fig. S11b). The increase in DRR incidence was also evident at 50% flowering and podding stages of chickpea growth (Supplementary Fig. S9c–f). Yield reduction in CS and PS was concomitant with increased disease incidence. The total average yield in the CS treatment was 63.36 gm/m^2^ compared to 110.86 gm/m^2^ in the PS treatment in location-1 during the 2018–2019 field trial (Supplementary Fig. S10a). A reduction in chickpea root volume was observed under the CS treatment (Supplementary Fig. S11). In addition, increased number of root tips and forks in chickpea was observed in LSM compared to control (Supplementary Fig. S11). Apart from chickpea genotype PUSA 372, which was used in all the field trial locations, increased DRR disease incidence was also observed for chickpea genotypes ICC4958 and JG 62 in a field trial (Supplementary Fig. S12). In all three chickpea genotypes, roots from CS treatment plots contained significantly higher number of *R. bataticola* microsclerotia compared to roots from the PS treatment (Fig. 5) showing that irrespective of their level of disease resistance, they are vulnerable to DRR under low soil moisture conditions. Increased DRR disease incidence was also observed in farmers’ fields with low or no irrigation in Anantapur (Supplementary Fig. S13), Kurnool, Kaddapa, and Sriganganagar districts in India (Supplementary Fig. S14) compared to well-irrigated fields.

## Discussion

DRR outbreak in fields can severely compromise chickpea production^6^. Therefore, the foresight of DRR disease occurrence is critical for limiting its effect on chickpea production. Early disease prediction can allow farmers to employ appropriate measures before infection or at the early stages of pathogen appearance as it is difficult to control DRR at later stages. DRR occurrence is highly influenced by environmental conditions for entry as well as its growth in plants. DRR in chickpea is known to thrive under low soil moisture and high-temperature conditions^5,8^. The hypothesis behind our current study was that DRR incidence in chickpea could be predicted in the field by understanding the influence of each environmental and edaphic factor involved in DRR disease progression. Based on the DRR disease incidence data from various public respurces, we report here that total rainfall and maximum temperature during the chickpea growing season largely impact DRR incidence in chickpea. Rainfall negatively influenced DRR incidence, whereas temperature positively regulated DRR incidence, and consequently, low rainfall and high temperature increased DRR disease incidence (Fig. 1). We also used a neural network-based simulation model with rainfall and temperature as input variables and predicted DRR incidence with an accuracy of 84%. Machine learning methods like ANN^25–28^ have been rigorously used to understand plant–pathogen–environment interactions and to identify the factors most relevant to disease incidence^14,15^. ANN has the advantage of establishing nonlinear relationships and is thus superior to linear regression and multiple linear regression^13^. In the current model, we used three environmental factors as inputs, among which the combination of rainfall and maximum temperature could most closely predict DRR incidence. To accommodate genotypic variation and the influence of different soil types in disease incidence, numerically converted genotype and soil details were incorporated as inputs along with environmental factors. When rainfall and temperature were used alone for training, the predicted DRR incidence were largely different from that of an actual incidence. The ANN-based prediction suggested that DRR incidence is highly dependent on the combination of rainfall and temperature.

Rainfall determines the soil moisture content of crop fields as both factors are linearly related^29^. Similarly, temperature majorly influences the moisture content of the soil in the field^30–32^. Thus, together, these two parameters decide the soil moisture level of the field. In the present study, we observed a high correlation between the average temperature of October and disease incidence, indicating that the low soil moisture due to high temperature during chickpea sowing and germination affects DRR incidence by either increasing the pathogen load in soil or facilitating pathogen entry into the plant during the early plant growth phase. In addition, the maximum temperature during the podding period, i.e., February, was highly correlated with DRR incidence. Again, high temperatures during podding can influence the fungus itself or alternatively decrease the soil moisture level by increasing evapotranspiration. Alternatively, high temperature may activate the pathogen in the soil, which otherwise is in the dormant phase for long period of time^33^. Further studies are required to understand this mechanism. Similar to the correlation results, ANN training with a combination of total rainfall from November to January and the average maximum temperature of October and November resulted in the best-fit model with high prediction accuracy. Also, similar to correlation analysis, ANN trained with the combination of total rainfall from November to January, and the average maximum temperature of February showed a high prediction accuracy. Both correlation and ANN training indicated a strong influence of the combination of the maximum temperatures for October and February with total rainfall from November to January in influencing DRR disease incidence.

A plethora of studies have examined plant–pathogen–water interactions. Usually, high moisture supports foliar disease development, while the influence of water in root disease varies with the pathogen and plant type^1,34^. *R. bataticola* infection and DRR disease development in chickpea have been reported to increase under low soil moisture^5,8,12,35^. However, the exact mechanism for the same has not been deciphered yet. Besides low soil moisture, many other environmental and edaphic factors interact with the plant and pathogen during DRR disease development, but their direct or indirect role in disease development or water deficit stress imposition has not been reported in the literature. Our field experiment at seven different geographical locations indicated the relation of low soil moisture in DRR development. In addition, we observed the relationships of P, K, and OC content of the soil, as well as soil type, with disease occurrence. Edaphic factors have been shown to influence root diseases in various ways^36^. The composition of the soil, depending on the percentage of clay, silt, or sand, decides the water-holding capacity of the soil, and in turn, decides the soil moisture content. Smaller particles like silt and clay hold more water due to a larger surface area. The present correlation analysis showed a positive correlation between DRR and clay content and a negative correlation between silt and DRR incidence. The negative correlation between silt and DRR can be inferred in terms of soil moisture content; however, the role of clay in DRR incidence is more than just in terms of water-holding capacity of the soil. Taya and group^12^ reported higher DRR incidence in chickpea grown on sandy soil and clay soil. They also reported increased DRR incidence with an increase in nitrogen (N) content and decreased DRR incidence with increased P content of the soil. However, we observed a positive correlation of P with DRR incidence in our study. A strong positive correlation between DRR incidence and soil K levels and OC was also observed in our study. Although the role of K in DRR incidence has not been reported earlier, for various other fungal pathogens, K^+^ deficiency has been found to increase fungal disease in plants^37^. Overall, the correlation analyses suggested important roles of nutrients like P, K, and OC in DRR incidence; therefore, maintaining adequate levels can help in controlling DRR incidence in chickpea.

In summary, this study unravels the dependence of DRR incidence in chickpea on the soil moisture content, with severe DRR occurring mainly under very low soil moisture. Simulation modeling suggested that rainfall and temperature majorly impact DRR incidence, probably via influencing soil moisture content or *R. bataticola* mycelial growth. Soil type and nutrients like K further influence DRR occurrence. We propose that detailed information on the role of edaphic factors on DRR incidence can be used to develop simulation methods for a more accurate prediction of DRR incidence in the farmers’ fields and thereby limit severe yield losses caused by this disease.

## Materials and methods

### Data acquisition for simulation modeling by ANN

Data for DRR incidence in the field were acquired from publicly available research articles and theses to perform ANN-based simulation modeling. In total, nine research articles and three Ph.D. theses that reported DRR incidence in the field across different geographical locations in India were collected (Supplementary Table S1). Data from all these sources were assembled with information such as location (including up to village level, if available), percent DRR incidence, soil type, irrigation pattern, and seed treatment (Supplementary File S1). The DRR disease incidence data from rainfed conditions were considered for the ANN training. The air temperature and rainfall data for the respective locations (from the closest weather stations) were obtained from the India Meteorological Department (IMD; https://mausam.imd.gov.in/). The monthly averages for maximum and minimum temperatures from October to February were calculated (Supplementary File S1). Similarly, total rainfall per month from October to February was calculated (Supplementary File S1).

### Steps involved in the execution of ANN

The steps followed for the ANN analyses are illustrated in Supplementary Fig. S1.

The neural network was performed in R using the neuralnet package^38^ (https://github.com/bips-hb/neuralnet). A combination of input variables (Supplementary Table S2) was used to study the closest set of weather variables influencing DRR disease incidence. The genotypes and soil types were converted to numeric codes for use as input values (Supplementary File S1). For all the neural network modeling, input, and output data were scaled using min–max normalization^39^.

$$MinMax\, scaled\,data=\frac{data -minimum\,of\,data}{range\,of\,data}$$

The dataset was further divided into 70% and 30% for training and testing, respectively. Training of the model was performed by varying the number of hidden layers and input variables. Neuralnet uses the backpropagation algorithm for network training^39^. The performance of the model was examined by DRR prediction for the test set. Next, k-fold cross-validation was performed with k = 10. The average of the coefficient of correlation (r) of all 10 networks was calculated, and the best-fit network with the highest average was selected for validation. The DRR disease incidence obtained by neuralnet was rescaled using the following formula^39^:

$$ {\text{Rescaled}}\,{\text{DRR}}\,{\text{DI = DRR}}\,{\text{DI}}\,{*}\,{\text{range}}\,{\text{of}}\,{\text{data + minimum}}\,{\text{of}}\,{\text{data}}{.} $$

The prediction efficiency between actual and predicted DRR for the test set was checked by calculating the correlation coefficient (r), coefficient of determination (R^2^), and root mean squared error (RMSE). Supplementary Table S2 summarizes the number of hidden layers, r, R^2^, and RMSE values for each combination of input variables.

DRR incidence was categorized into LowDRR (DRR incidence < 30%) and HighDRR (DRR incidence > 30%), and a confusion matrix was created with LowDRR as TN and HighDRR as TP. Prediction accuracy was checked using the following formula:

$$Accuracy=\frac{TP+TN}{TP+FN+TN+FP},$$

where TP = true positive, TN = true negative, FN = false negative, FP = false positive.

A dataset containing DRR disease incidence and respective input weather data from field location-4 and experimental data from Sinha et al.^5^ from location-2 (Supplementary File S1) was used for the validation of best-fit ANN models. Prediction accuracy was determined with r, R^2^, and RMSE and checked with a confusion matrix.

### Field experiments for assessing DRR incidence and associated factors

Field experiments to study the impact of environmental and edaphic factors on DRR disease incidence were conducted at three different locations during the year 2018–2019 and seven different locations during the year 2019–2020 (Supplementary Fig. S3). *R. bataticola* pathogen load in each field was checked by assessing a pathogen-susceptible chickpea varieties JG62, BG212, JAK9218, and L550. Pathogen load was categorized into mild, moderate, and severe, depending on percent DRR disease incidence (Supplementary Table S4). To study the impact of soil moisture on DRR incidence, four types of treatment plots with two different irrigation regimes with fungicide-treated plots and untreated plots were maintained. For fungicide treated plot seed were treated with a mixture of Bavistin (50% WP Carbendazim, Hindustan Antibiotics Limited, Pune) and SAAF (Carbendazim 12% Mancozeb 63% WP, United Phosphorus Limited, Mumbai) in the concentration of 10 g/kg of seeds before sowing. Adequately irrigated plots (to maintain soil moisture of 15–25%) with fungicide treatment were considered as control plots. Less irrigated plots (to keep the soil moisture between 5 and 12% for the most duration) with fungicide treatments were considered as low soil moisture treatment plots. Similarly, adequately irrigated plots with no fungicide treatment were considered pathogen treatment plots, while less irrigated plots with no fungicide treatment were named combined stress treatment plots. Four replicates were maintained for each treatment, and they were randomized using a randomized complete block design (RCBD) (Supplementary Fig. S4). Soil moisture was recorded using a Leutron soil moisture meter (PMS-714; Leutron Electronics, Taipei, Taiwan). Data for air temperature, humidity, and rainfall were obtained from India Meterological Department. Percent disease incidence data were recorded as per the method mentioned in Sinha et al.^5^ (Supplementary File S1). Total seed weight was recorded at the end of the field trial (Supplementary File S1). The disease severity index was calculated with five disease scores (Supplementary Fig. S11) using the formula mentioned below^40^:

$$ DSI = \sum { }\left( {{\text{Class frequency }} \times {\text{ score of rating class}}} \right)/\left( {\text{Total number of observations}} \right){ } \times \left( {\text{maximal disease index}} \right)]{ } \times { }100 $$

### Assessment of soil properties and composition

Soil samples collected from field locations were analysed for soil N, P, K, OC, pH, EC, soil type, and water-holding capacity at the Central Laboratory for Soil and Plant Analysis, Division of Soil Science and Agricultural Chemistry, Indian Agricultural Research Institute, Pusa, New Delhi.

### Field data analyses

Averages (with the standard error of the mean) of monthly soil moisture, air temperature, air humidity, and rainfall were calculated and presented for each location. For the DRR severity index (DSI) and DRR disease incidence, the average of four RCBD replicates was calculated. Significant differences between means were tested by Student’s *t*-test and one-way ANOVA with Tukey’s post hoc test. Pearson’s correlation analyses were performed between DRR disease incidence and each of the weather and edaphic parameters. All the statistical analyses were performed in R.

### Microscopy observations

Plant roots were collected from Pusa 372, JG 62 (aka. ICC 4951) and ICC 4958 genotypes during the crop maturation period and hand sections at 2 cm from the point of seed attachment were made using blade (Gillette, Mumbai, India). Sections were observed for the presence for microsclerotia in the epidermis, cortex, and vascular bundles and pith regions. Sections were observed under 0.5X objective lens of research stereo microscope SMZ25 (Nikon Corporation, Tokyo, Japan).

### Statement on third party material

Root samples were collected from our laboratory experiments from the said varieties (under ‘Microscopy observations’ above). The plants were grown in our growth chamber and after the said treatments the roots were taken. Since it is our own experiments in the lab, there will be no permission needed.

## Supplementary Information

Supplementary Information 1.

Supplementary Information 2.

## References

[1] Bostock RM, Pye MF, Roubtsova TV (2014). Predisposition in plant disease: Exploiting the nexus in abiotic and biotic stress perception and response. Annu. Rev. Phytopathol..

[2] Suzuki N, Rivero RM, Shulaev V, Blumwald E, Mittler R (2014). Abiotic and biotic stress combinations. New Phytol..

[3] Kissoudis C (2016). Responses to combined abiotic and biotic stress in tomato are governed by stress intensity and resistance mechanism. J. Exp. Bot..

[4] Pandey P, Irulappan V, Bagavathiannan MV, Senthil-Kumar M (2017). Impact of combined abiotic and biotic stresses on plant growth and avenues for crop improvement by exploiting physio-morphological traits. Front. Plant Sci..

[5] Sinha R, Irulappan V, Mohan-Raju B, Suganthi A, Senthil-Kumar M (2019). Impact of drought stress on simultaneously occurring pathogen infection in field-grown chickpea. Sci. Rep..

[6] Nene, Y. L., Reddy, M. V., Haware, M. P. & Ghanekar, A. M. *Field Diagnosis of Chickpea*. **28** (2012).

[7] Bhatti MA, Kraft JM (1992). Influence of soil moisture on root rot and wilt of chickpea. Plant Dis..

[8] Sharma M, Pande S (2013). Unravelling effects of temperature and soil moisture stress response on development of dry root rot [Rhizoctonia bataticola (Taub.)] Butler in Chickpea. Am. J. Plant Sci..

[9] Ghosh R, Sharma M, Telangre R, Pande S (2013). Occurrence and Distribution of Chickpea Diseases in Central and Southern Parts of India. Am. J. Plant Sci..

[10] Wagh, P. Studies on dry root rot (Rhizoctonia bataticola Taub (Butler)) of chickpea (Cicer arietinum). (2015).

[11] Srinivas, P. Studies on dry root rot [Rhizoctonia bataticola (Taub.) Butler] of chickpea (Cicer arietinum L.). (2016).

[12] Taya, R. S., Tripathi, N. N. & Panwar, M. S. Influence of soil type, soil moisture and fertilizers on the severity of chickpea dry root-rot caused by Rhizoctonia bataticola (Taub.) Butler. *Indian J. Mycol. Plant Pathol.* **18**, 133–136 (1988).

[13] Schmidhuber J (2015). Deep Learning in neural networks: An overview. Neural Netw..

[14] Kaundal R, Kapoor AA, Raghava GPS (2006). Machine learning techniques in disease forecasting: A case study on rice blast prediction. BMC Bioinformatics.

[15] Kim Y, Roh JH, Kim HY (2017). Early forecasting of rice blast disease using long short-term memory recurrent neural networks. Sustain..

[16] Malicdem, A. R. Rice Blast Disease Forecasting for Northern Philippines Rice Blast Disease Forecasting for Northern Philippines College of Information Technology Department of Information Systems and Computer Science. (2015).

[17] De Wolf ED, Francl LJ (2000). Neural network classification of Tan spot and Stagonospora blotch infection periods in a wheat field environment. Phytopathology.

[18] Paul PA, Munkvold GP (2005). Regression and artificial neural network modeling for the prediction of gray leaf spot of maize. Phytopathology.

[19] Panigrahi, K. P., Das, H., Sahoo, A. K. & Moharana, S. C. Maize Leaf Disease Detection and Classification Using Machine Learning Algorithms. in *Progress in Computing, Analytics and Networking* (eds. Das, H., Pattnaik, P. K., Rautaray, S. S. & Li, K.-C.) 659–669 (Springer Singapore, 2020).

[20] Kitchen NR, Drummond ST, Lund ED, Sudduth KA, Buchleiter GW (2003). Soil electrical conductivity and topography related to yield for three contrasting soil-crop systems. Agron. J..

[21] Miao Y, Mulla DJ, Robert PC (2006). Identifying important factors influencing corn yield and grain quality variability using artificial neural networks. Precis. Agric..

[22] Norouzi M, Ayoubi S, Jalalian A, Khademi H, Dehghani AA (2010). Predicting rainfed wheat quality and quantity by artificial neural network using terrain and soil characteristics. Acta Agric. Scand. Sect. B Soil Plant Sci..

[23] Ayoubi S, Sahrawat KL (2011). Comparing multivariate regression and artificial neural network to predict barley production from soil characteristics in Northern Iran. Arch. Agron. Soil Sci..

[24] Adisa OM (2019). Application of artificial neural network for predicting maize production in South Africa. Sustain..

[25] De Wolf ED, Francl LJ (2000). Neural network classification of Tan spot and Stagonospora blotch infection periods in a wheat field environment. Phytopathology.

[26] Chakraborty S (2004). Weather-based prediction of anthracnose severity using artificial neural network models. Plant Pathol..

[27] Sharma, P., Singh, B. K. & Singh, R. P. Prediction of potato late blight disease based upon weather parameters using artificial neural network approach. *2018 9th International Conference on Computing and Communication Network Technology ICCCNT 2018* 1–13 (2018). 10.1109/ICCCNT.2018.8494024

[28] Paul PA, Munkvold GP (2005). Regression and artificial neural network modeling for the prediction of gray leaf spot of maize. Phytopathology.

[29] Varikoden H, Revadekar JV (2018). Relation between the rainfall and soil moisture during different phases of Indian monsoon. Pure Appl. Geophys..

[30] Lakshmi V, Jackson TJ, Zehrfuhs D (2003). Soil moisture-temperature relationships: Results from two field experiments. Hydrol. Process..

[31] Jin MS, Mullens T (2014). A study of the relations between soil moisture, soil temperatures and surface temperatures using ARM observations and offline CLM4 simulations. Climate.

[32] Feng H, Liu Y (2015). Combined effects of precipitation and air temperature on soil moisture in different land covers in a humid basin. J. Hydrol..

[33] Srinivas P, Ramesh Babu S, Sharma M, Narayan Reddy P, Pushpavathi B (2017). Effect of temperature on Rhizoctonia bataticola and dry root rot in chick pea. Int. J. Curr. Microbiol. Appl. Sci..

[34] Cook RJ, Papendick RI (1972). Influence of water potential of soils and plants on root disease. Annu. Rev. Phytopathol..

[35] Jooste, W. J. Infection of crops by Rhizoctonia bataticola as influenced by soil moisture. 15–18 (1969).

[36] Huber, D., Römheld, V. & Weinmann, M. Relationship between Nutrition, Plant Diseases and Pests. *Marschner’s Miner. Nutr. High. Plants Third Ed.* 283–298 (2011). 10.1016/B978-0-12-384905-2.00010-8

[37] Walters DR, Bingham IJ (2007). Influence of nutrition on disease development caused by fungal pathogens: Implications for plant disease control. Ann. Appl. Biol..

[38] Fritsch, S., Guenther, F., Wright, M.N., Suling, M. & Mueller, S. M. *Package ‘neuralnet’ Training of Neural Networks* (2019).

[39] Ciaburro, G. & Venkateswaran, B. *Neural network with R*. *Packt* **91**, (Packt Publishing, 2017).

[40] Chiang, K. S., Liu, H. I., Tsai, J. W., Tsai, J. R. & Bock, C. H. A discussion on disease severity index values. Part II: using the disease severity index for null hypothesis testing. *Ann. Appl. Biol.* (2017). 10.1111/aab.12396

