# Supplementary material for: Low soil moisture predisposes field-grown chickpea plants to dry root rot disease: evidence from simulation modeling and correlation analysis
Source: Sci Rep. 2021 Mar 22;11:6568. doi: 10.1038/s41598-021-85928-6 (PMC7985499; doi:10.1038/s41598-021-85928-6)
Supplement: Supplementary file 1 — Supplementary Information 1. [file 41598_2021_85928_MOESM1_ESM.pdf]

Supplementary material

Supplementary figures 1-14
Supplementary tables 1-4

Low soil moisture stress predisposes field-grown chickpea plants to dry root rot disease: Evidence from simulation modeling and correlation analysis

Ranjita Sinha[1], Vadivelmurugan Irulappan[1], Basavanagouda S. Patil[2], Puli Chandra Obul Reddy[3], Venkategowda Ramegowda[4], Basavaiah Mohan-Raju[4], Krishnappa Rangappa[5], Harvinder Kumar Singh[6], Sharad Bhartiya[7], Muthappa Senthil-Kumar[1,*]

[1]National Institute of Plant Genome Research, Aruna Asaf Ali Marg, New Delhi-110067, India
[2]ICAR-IARI-Regional Research Center, P. B. Road, Dharwad- 580001, India
[3]Department of Botany, Yogi Vemana University, Kadapa, Andhra Pradesh- 516005, India
[4]Department of Crop Physiology, University of Agricultural Sciences, GKVK, Bangalore- 560 065 India
[5]Division of Crop Production, ICAR Research Complex for North Eastern Hill Region,Umroi Road, Umiam -793103 India
[6]Department of Plant Pathology, Indira Gandhi Krishi Vishwavidyalaya, Raipur- 492012 India
[7] Department of Chemical Engineering, Indian Institute of Technology Bombay, Powai, Mumbai- 400076, India

*corresponding author: skmuthappa@nipgr.ac.in
Muthappa Senthil-Kumar
National Institute of Plant Genome Research,
Aruna Asaf Ali Marg,
P.O. Box No. 10531
New Delhi - 110 067

Supplementary Figure S1

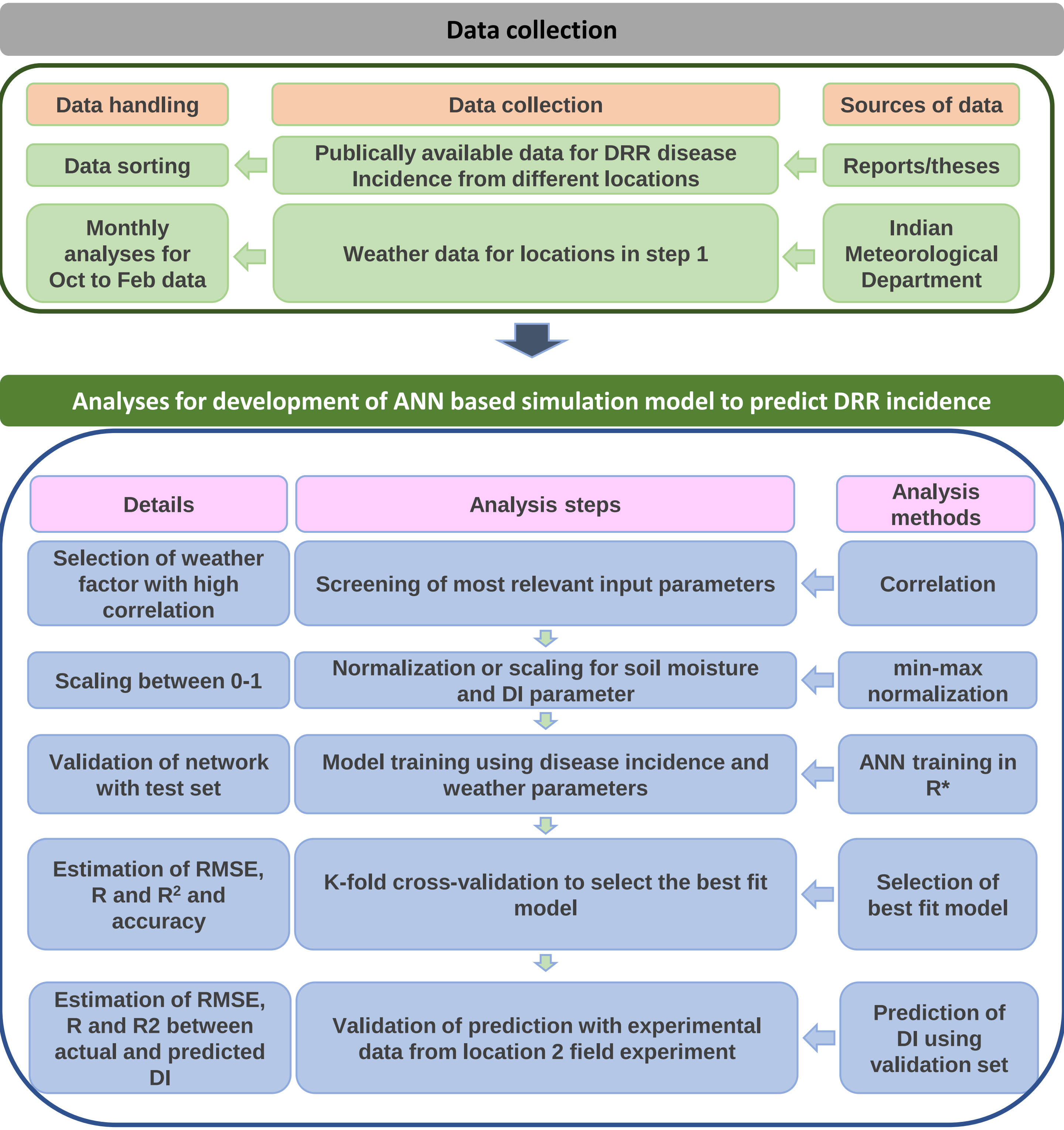

Supplementary Figure S1. Overview of the pipeline for Artificial Neural Network-based simulation modelling for dry root rot disease prediction in chickpea

A methodology illustrated in this figure was adopted to establish the relation of weather factors (rainfall and temperature) with DRR disease incidence and to develop a method to predict DRR incidence across locations and years. DRR incidence data in the field for various locations were acquired from research articles and theses. The data for location (up to village level), previously grown crop, year, soil type, irrigated/not-irrigated, and percent DRR disease incidence were retrieved from the literature (Supplementary File S1). The weather data for the locations and year mentioned in the public resources were obtained from the India Meteorological Department (IMD, https://mausam.imd.gov.in/). Weather data for the rabi season (October to February) were analysed. The total monthly rainfall and average monthly temperature were calculated for the October to February (Supplementary File S1). Further, the total of bimonthly and trimonthly for rainfall and average temperature for the same period were calculated (Supplementary file S1). Before proceeding with the development of Artificial Neural Network (ANN) based simulation modelling tool for the prediction of DRR disease incidence in chickpea, Pearson correlation was checked. This was done among DRR disease incidence, monthly, two-months, three-months total rainfall, an average of monthly minimum and maximum temperature . A correlation matrix was developed in R. The data set chosen for the ANN training was scaled using min-max normalization method to scale the data between 0-1. MinMax normalization method uses following formula, $scaled\ data = \frac{data - minimum\ of\ data}{range\ of\ data}$

For ANN training neuralnet algorithm in R was used. Neuralnet uses a backpropagation algorithm with a linear activation function by default. Different hidden layers were tried during training to select the most suitable hidden layer for the best fit. The entire dataset (of 96 datapoint) for the neural network training was divided into 70% training set (67 data points) and 30% testing set (29 data points). Prediction accuracy of the neural network was tested with calculated root mean square error (RMSE), coefficient of regression (r), and coefficient of determination (R^2) between actual DRR disease incidence of the testing set and predicted disease incidence for the testing set. Further, K-fold cross validation was performed to select the best fit models. Further, the neural network was validated with the validation data set. For the validation data set, DRR disease incidence, and respective rainfall and temperature data from the field trial conducted at Location 4 in current field trial and location 2 from Sinha et al. (2019) was used. Prediction accuracy was again checked using RMSE, r, and R^2. further, DRR incidence was divided into LowDRR (DRR incidence<30%) and HighDRR (DRR incidence>30%) and confusion matrix was created. Prediction accuracy was checked using following formula.

$$Accuracy = (TP + TN)/(TP + FN + TN + FP)$$

TP = Ture positive, TN = True negative, FN = false negative, FP = false positive

Sinha, R., Irulappan, V., Mohan-Raju, B., Suganthi, A., & Senthil-Kumar, M. (2019). Impact of drought stress on simultaneously occurring pathogen infection in field-grown chickpea. Scientific reports, 9(1), 1-15.

Supplementary Figure S2

a

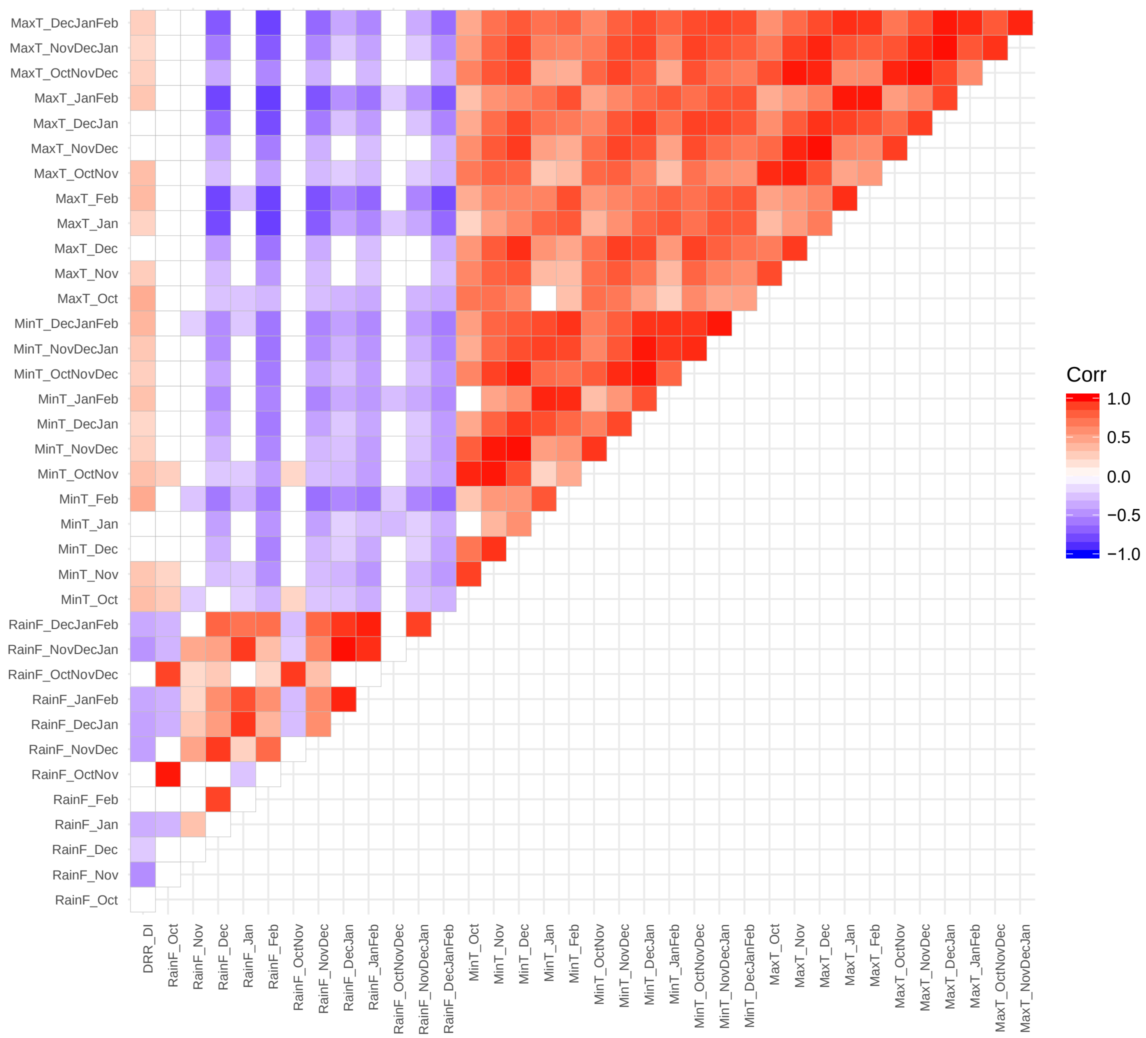

Supplementary Figure S2

b

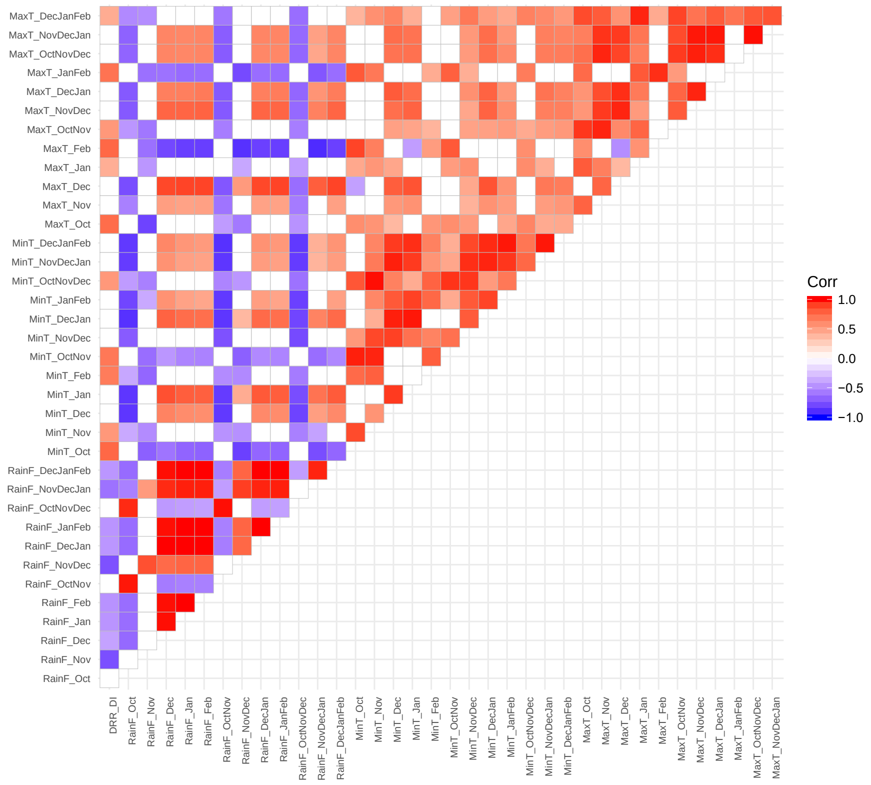

c

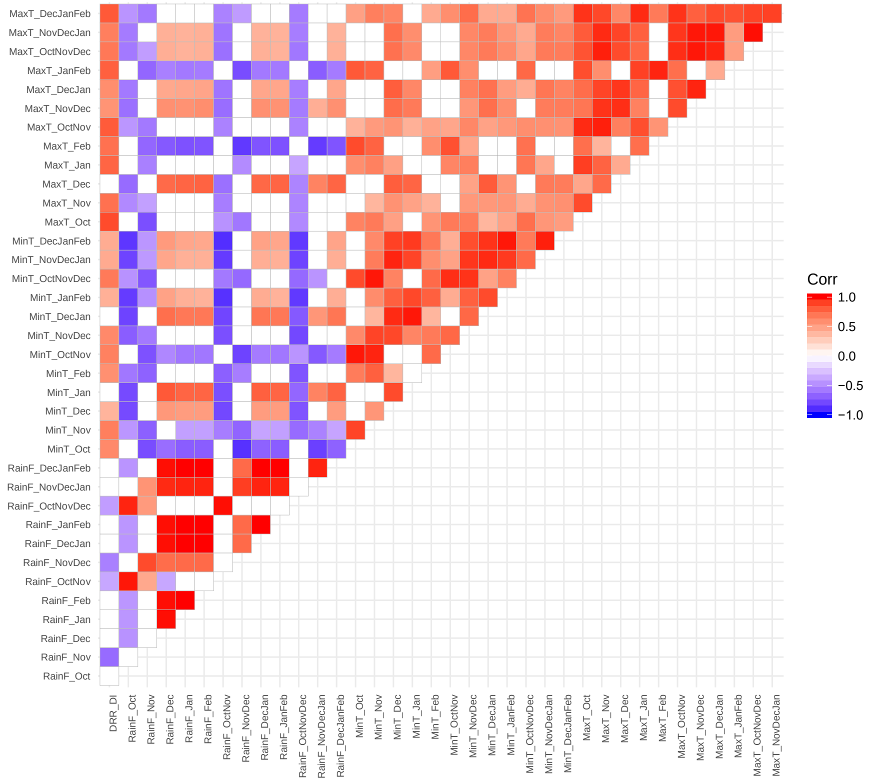

Supplementary Figure S2

d

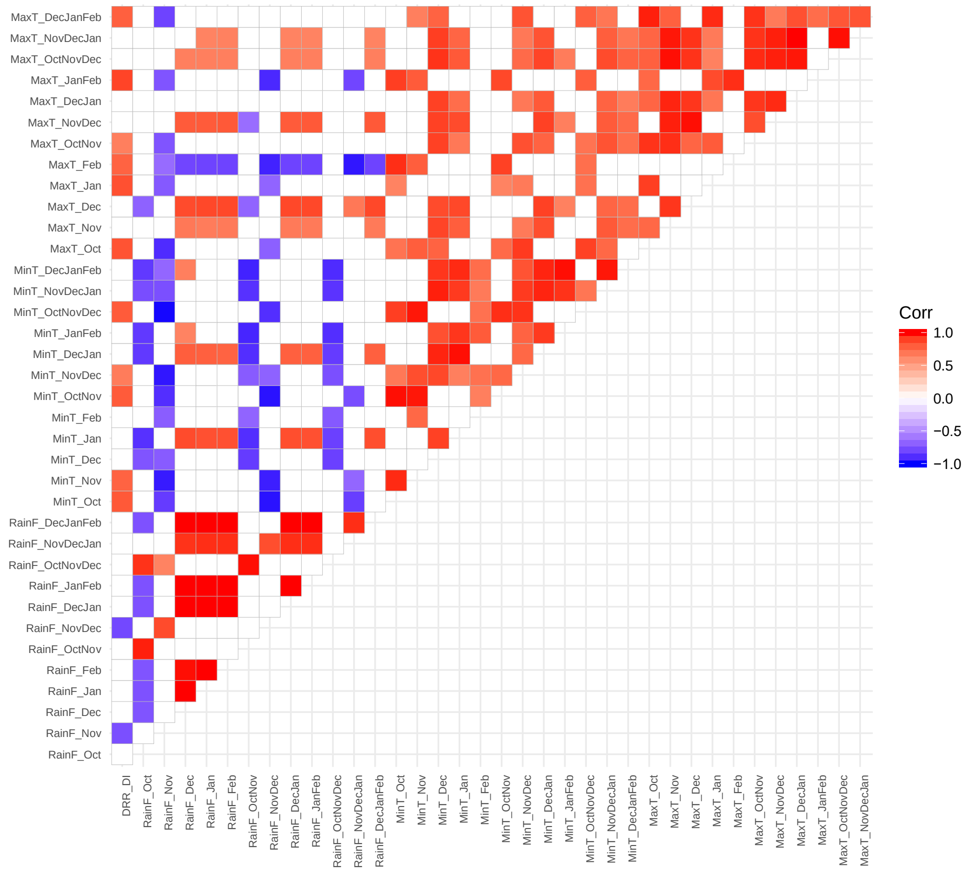

e

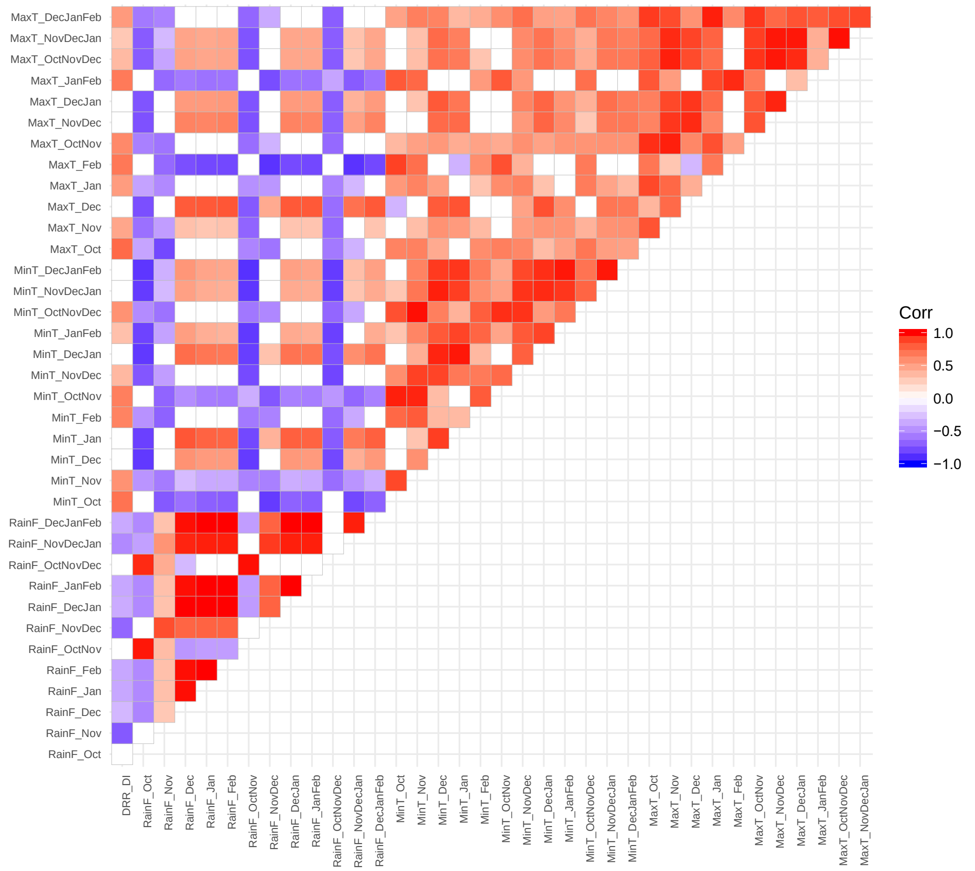

Supplementary Figure S2. Correlation analyses between DRR disease incidence and rainfall and temperature for different genotypes and soil types. Correlation analyses were performed with publicly available DRR disease incidence data to study the relation between DRR disease incidence and weather components rainfall and temperature/impact of weather factors on DRR disease incidence. DRR disease incidence data was gathered from published research articles and theses (Supplementary File S1). Weather data for the respective locations were acquired from IMD (https://mausam.imd.gov.in/).). Weather data for the field trial season from October to February for the specified year was considered, and the monthly average from October to February was calculated. A correlation matrix developed using Pearson's correlation method. The graph from (a) to (e) is showing the correlation between DRR disease incidence and weather parameters in complete data set (a), in dataset with genotype A1 (b) and dataset having genotype GJ11(c), in data set with only red soil (d), in black soil containing dataset (e). Red boxes represent a significant positive correlation, and blue boxes represent a significant negative correlation. Empty boxes represent nonsignificant correlation. Correlation with $p<0.05$ was taken as statistically significant. A negative correlation exists between DRR disease incidence and rainfall during November month (RainF_Nov), and a positive correlation exists between disease incidence and minimum and maximum temperature during October month (MinT_Oct, MaxT_Oct). October month is the sowing month of chickpea, and thus the positive correlation with the disease incidence indicates the indirect impact of temperature on disease incidence by probably influencing the soil moisture status.

RainF = rainfall, MinT = minimum temperature, MaxT = Maximum temperature, DRR DI = DRR disease incidence, Oct = October, Nov = November, Dec = December, Jan = January, Feb = February, Mar = March.

Supplementary figure S3

a

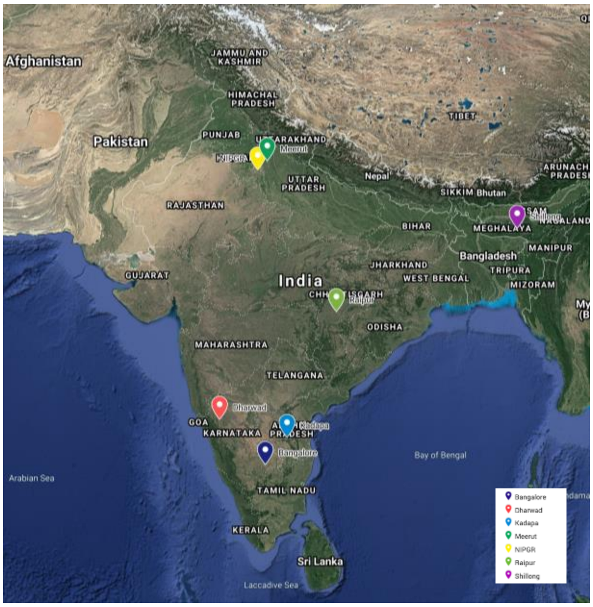

b

S. No.	Locations of trial	State	Location	Lat/Long	Average Temp*	Average Rainfall*	Soil Type
1	Location 1	New Delhi	NIPGR	28.61/77.20	25.2 °C	693 mm	Clay
2	Location 2	Karnataka	Bangalore	12.97/77.59	23.6 °C	831 mm	Red loamy soil/Laterite soil
3	Location 3	Uttar Pradesh	Meerut	28.98/77.70	24.7 °C	933 mm	Sandy Loam/Loam
4	Location 4	Karnataka	Dharwad	15.45/75.00	24.3 °C	885 mm	Black
5	Location 5	Meghalaya	Umiam	25°67/92°92	13.1 °C	2450 mm	Sandy to clayey loam-Alluvial soil
6	Location 6	Andhra Pradesh	Kadapa	14.54/78.59	29.2 °C	753 mm	Black
7	Location 7	Chhattisgarh	Raipur	21.25/81.62	26.8 °C	1276 mm	Red/black-Ferralitic soil

Supplementary Figure S3. Map showing the field trial locations in India. The field trial was conducted at seven different geographical locations named as Location 1-7 in six different states of India (**a and b**). Out of seven locations, two were in northern part of India, three in the southern part, one each in central and north-eastern part of India. The seven locations cover five agroclimatic zones of India. Location 1 and Location 3 was in arid western plains and North-Western Plains respectively. Location 2 was in Eastern Dry Zone and Location 4 was in Northern Dry Zone. Location 5 was in Sub tropical Hill, Location 6 belongs to Scarce rainfall zone of Rayalseema, and Location 7 was in C.G. Plains. The treatments comprised of low soil moisture (LSM), pathogen stress (PS), combined stress (CS), and control in four replicates in RCB design. The treatment details are provided in **Supplementary Figure S4**. The variation in average temperature, rainfall, and soil type was considered (b) while performing the field experiment to generalize our hypothesis. The map was prepared in Google My Maps (mymaps.Google.co.in). *Data collected from climate-data.org (https://en.climate-data.org/)

Supplementary figure S4

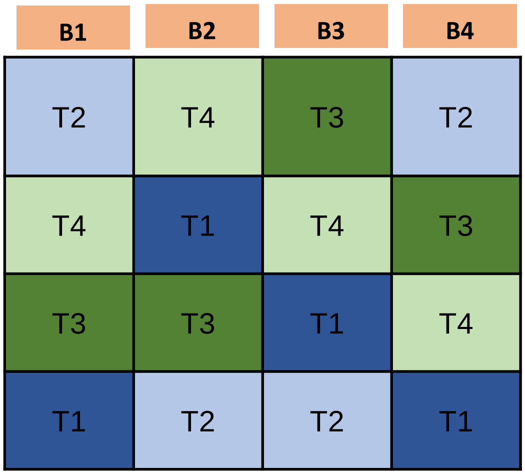

T1= Control, frequent irrigation to maintain 12-15% soil moisture + Fungicide

T2= low soil moisture (LSM), frequent irrigation to maintain 2-5% soil moisture + Fungicide

T3= Pathogen (PS), frequent irrigation to maintain 12-15% soil moisture + No Fungicide

T4= Combined stress (CS), frequent irrigation to maintain 2-5% soil moisture + No Fungicide

Supplementary Figure S4. Outline showing the treatments and statistical design used in the field experiments and RCB design . Three different treatments, namely low soil moisture (LSM), pathogen (PS) and combined stress (CS) were considered along with control for field trial at all the locations mentioned in **Supplementary Figure S3**. A plot size of 2x2 m^2 were sown with chickpea variety PUSA 372 at distance of 10 cm within rows and distance between rows were 30cm. Treatments were randomized using RCB design. A distance of around half meter were maintained between plots to avoid the percolation of water from irrigated plots to neighbouring plots. Two different irrigation regime were followed for control/PS and LSM/CS respectively. The soil moisture in control and PS plots were maintained between 12-20% while 2-5%were maintained in LSM and CS plots. Seeds for the control and LSM treatment plots were treated with fungicide Bavistin (10gm/kg of seeds) and SAAF (10gm/kg of seeds) in 1:1 ratio. Bavistin and SAAF in field (2kg/ha of each) to control DRR occurrence. All the field plots in all the locations were sick and the presence of *Rhizoctonia bataticola* was confirmed by uprooting and culturing infected chickpea root tissue (**Supplementary Figure S5**). Randomization of treatments in RCB was performed in R using **design.rcbd** from **agricolae** library (Mendiburu and Simon, 2015)**.**

De Mendiburu F, Simon R. 2015. Agricolae - Ten years of an open source statistical tool for experiments in breeding, agriculture and biology. PeerJ PrePrints 3:e1404v1

Supplementary figure S5

a Location 1

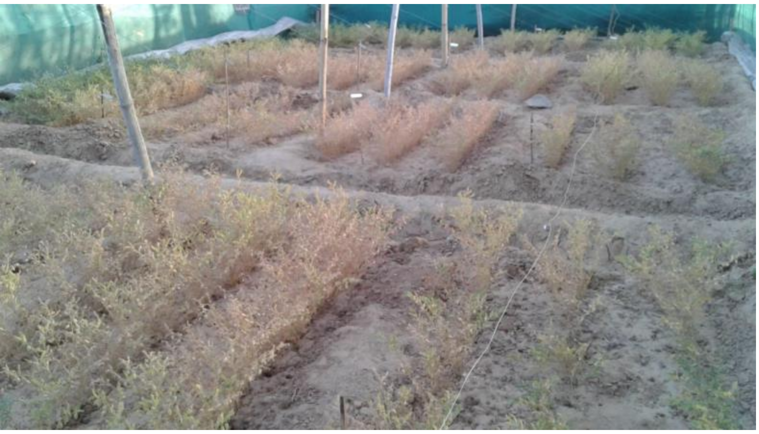

b Location 2

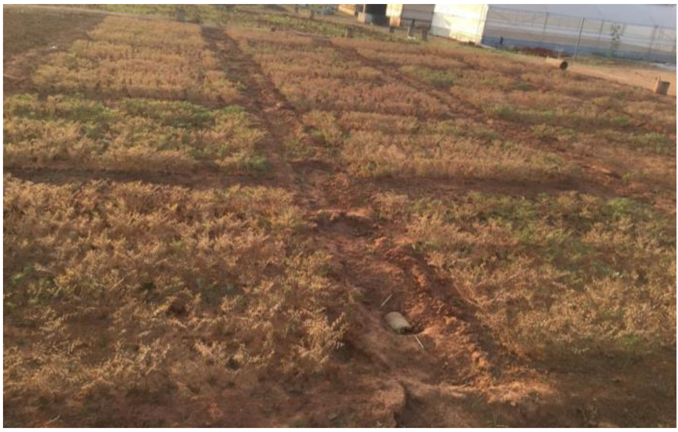

c Location 4

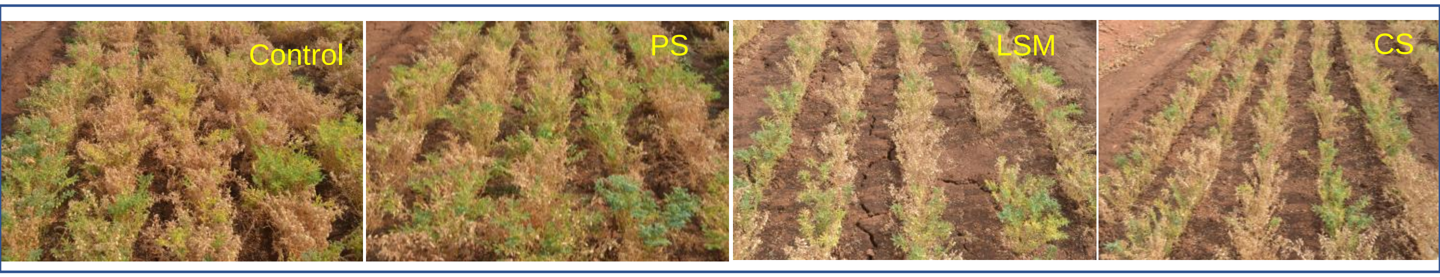

d Location 5

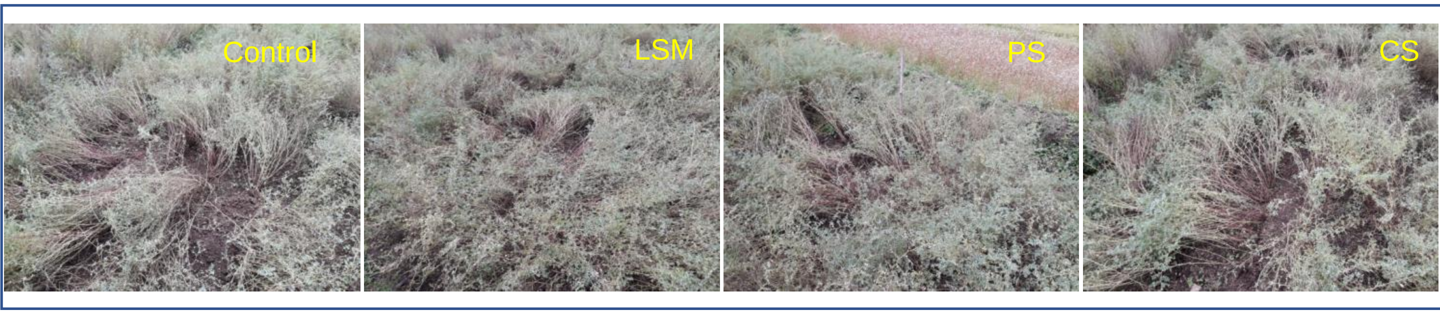

e Location 7

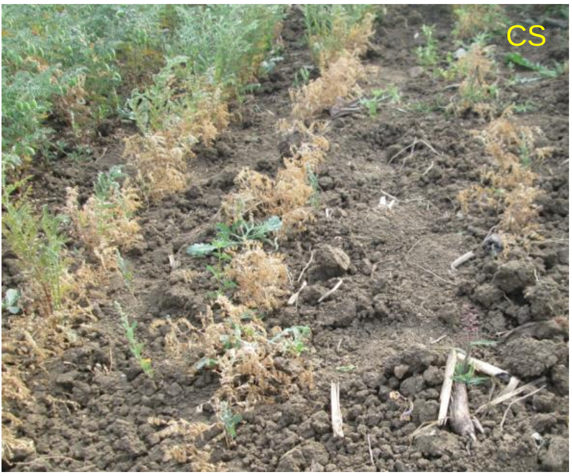

Supplementary Figure S5. Field view of different locations showing the DRR disease. The DRR diseased plants observed are represented here for Location 1 (**a**), Location 2 (**b**), Location 4 (**c**), Location 5 (**d**) and Location 7 (**e**). The first two panels (a) and (b) (location 1-2) are a field view as per the treatment design. The location 7 field view (e) is displaying the best DRR symptoms that appeared in the CS plot. LSM, low soil moisture; PS, Pathogen stress; CS, combined stress

Supplementary figure S6

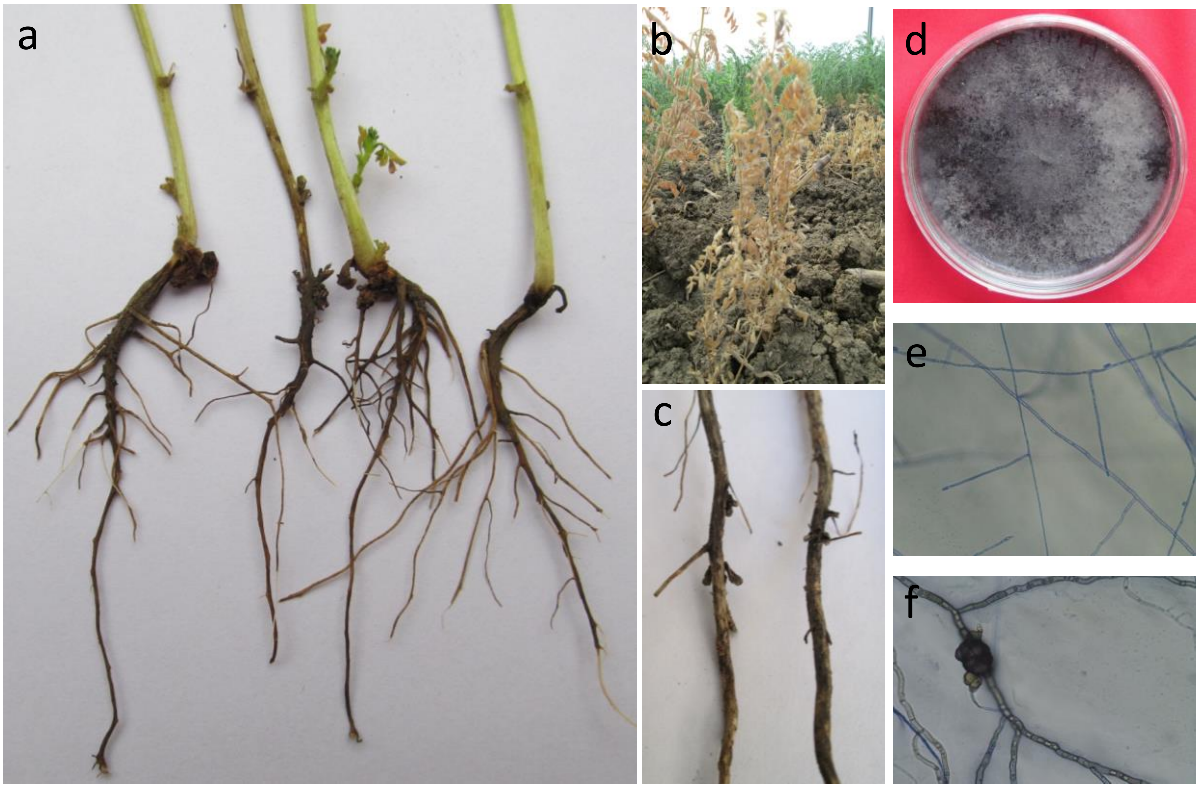

Supplementary Figure S6. DRR symptoms and fungal growth details as picturized from field trial plot.

Figure (a) represents DRR disease in chickpea root at an earlier stage of infection. Plants is the Figure (a) were uprooted two months post sowing during 50% flowering stage. Chickpea plants with foliar DRR disease symptoms (b) were uprooted and infected root (three months old plants, pod formation stage) with DRR symptoms (brittle roots and blackening of roots, less developed secondary roots) (c) were cultured to confirm the disease. Pure culture of *Rhizoctonia bataticola* (d) confirmed the DRR disease in the root. Further, Right-angled branching of *R. bataticola* (e) and formation of *R. bataticola* microsclerotia (f) confirmed the disease.

Supplementary figure S7

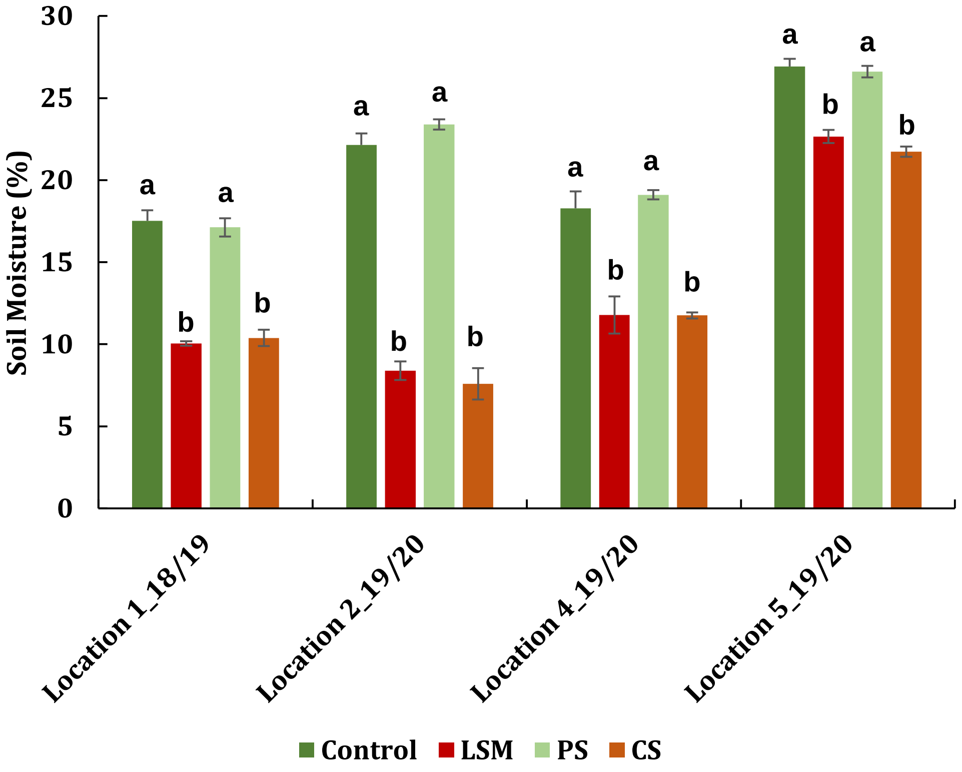

Supplementary Figure S7. Soil moisture data observed during the field trials. Soil moisture for locations were measured using Lutron PMS-714 moisture meter. Average of four block replicates of percent soil moisture data with SEM for Location 1 for trial year 2918-19 and locations 2, 4 and 5 for the trial year 2019-20 are represented in the graph. The soil moisture data is an average of four RCB replicates. Statistical significance difference between means is checked by one way ANOVA. The different letter denotes a significant difference between mean at $p<0.05$. LSM, low soil moisture; PS, Pathogen stress; CS, combined stress

Supplementary figure S8

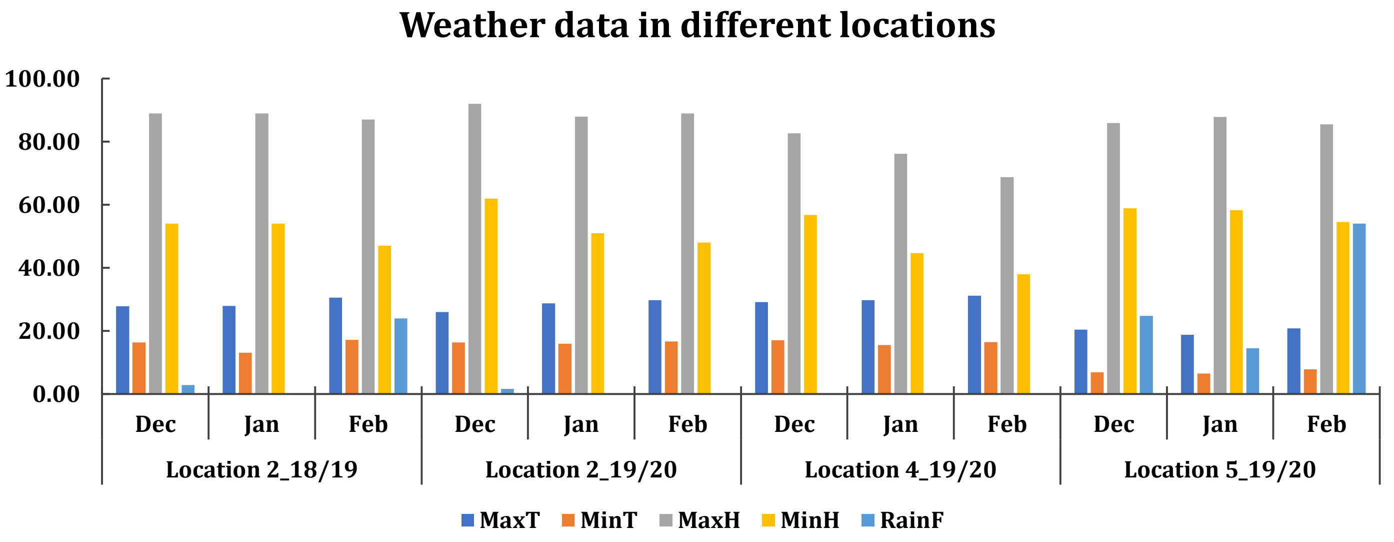

Supplementary Figure S8. Monthly average of air temperature, humidity, and rainfall data observed during the field trials. The data for the daily minimum temperature in ºC (MinT), maximum temp in ºC (MaxT), rainfall in mm (RainF), percent maximum humidity (MaxH) and percent minimum humidity (MinH) were acquired for the months from December (Dec) to February (Feb) for the specific years 2018-19 and 2019-20 from the Agrometeorology department of the respective locations. The monthly average of all the weather factors from location 2 for year 2018-19 and for location 2, location 4 and location 5 for the year 2019-20 are shown in bar graph.

Supplementary figure S9

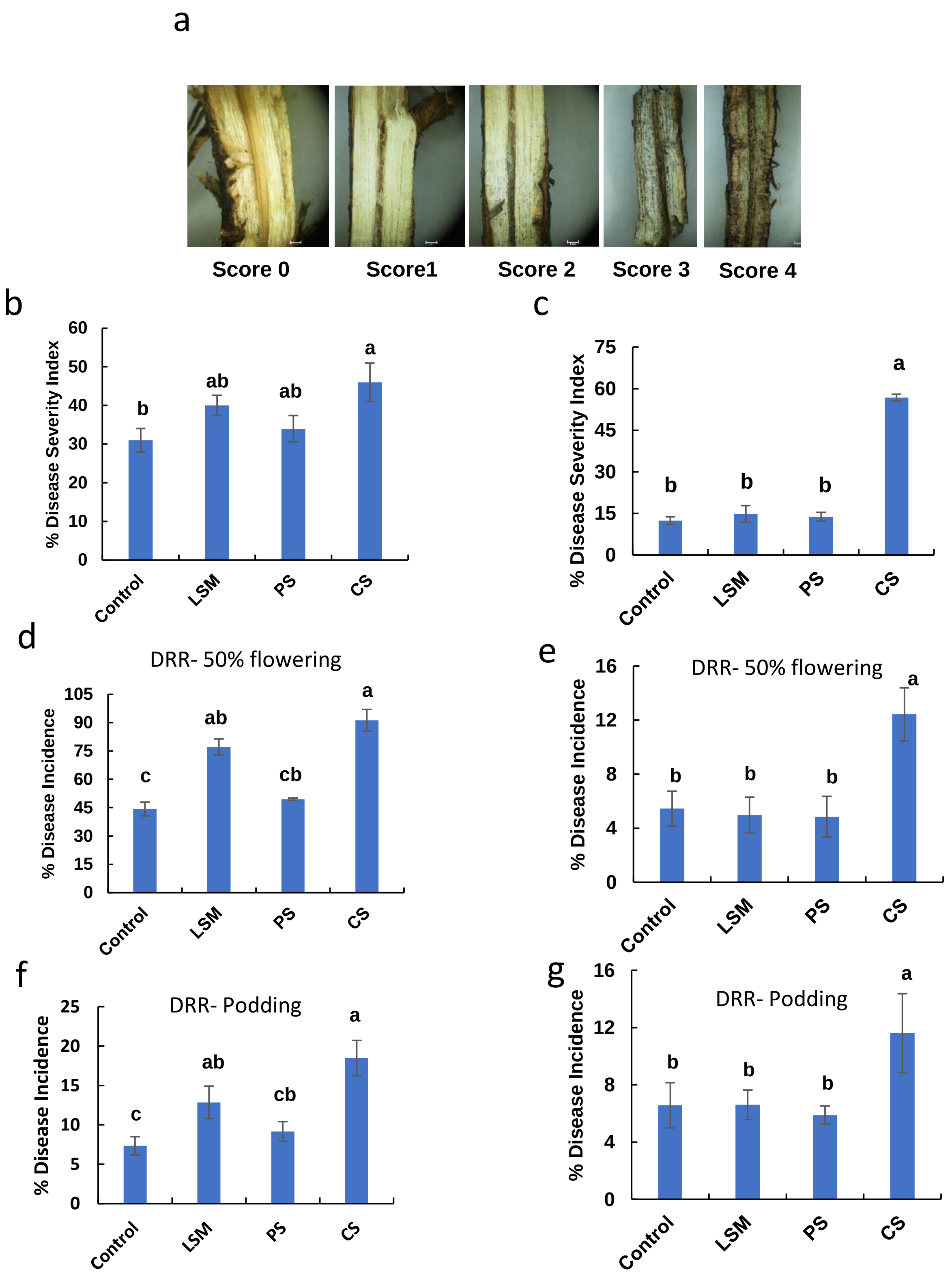

Supplementary Figure S9. Dry root rot disease incidence under various growth stage in field trials. Disease severity index was calculated with five disease score (a) using the below mentioned formula and scoring scheme. Dry root rot (DRR) disease severity index was calculated for all the treatments in all the field trial locations 4 (b) and Location 5 (c). Disease incidence was calculated at 50% flowering stage at location 4 (d) and Location 5 (e), and further at podding stage for Location 4 (f) and Location 5 (g) using the below mentioned formula. Both the DRR severity index (DSI) and DRR disease incidence is average of four RCB replicates. Statistical significance difference between means are checked by One Way ANOVA and Tukey's Posthoc test. Different letter denotes significant difference between mean at $p<0.05$. LSM, low soil moisture; PS, Pathogen stress; CS, combined stress

DSI = ∑ (Class frequency × score of rating class)/(Total number of observations) × (maximal disease index)] × 100

% Disease Incidence= (Number of infected plants/total number of plants)X100.

Supplementary figure S10

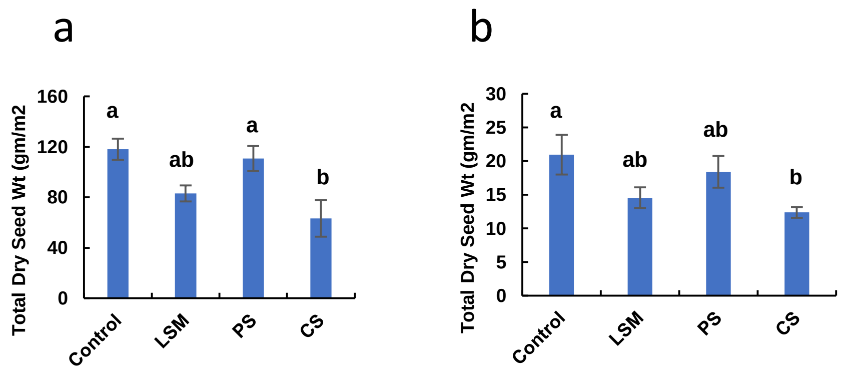

Supplementary Figure S10. Total yield under different treatments in different year/location field trials. Total yield was calculated for field trial year 2018-19 for Location 1 and trial year 2019-20 for Location 4 is represented in graphs (a) and (b), respectively. Each bar for yield in the graph is the average of four RCB replicates with SEM as an error bar. Statistical significance difference between means is checked by one way ANOVA and Tukey's Posthoc test. The different letters denotes a significant difference between mean at $p<0.05$. LSM, low soil moisture; PS, Pathogen stress; CS, combined stress

Supplementary figure S11

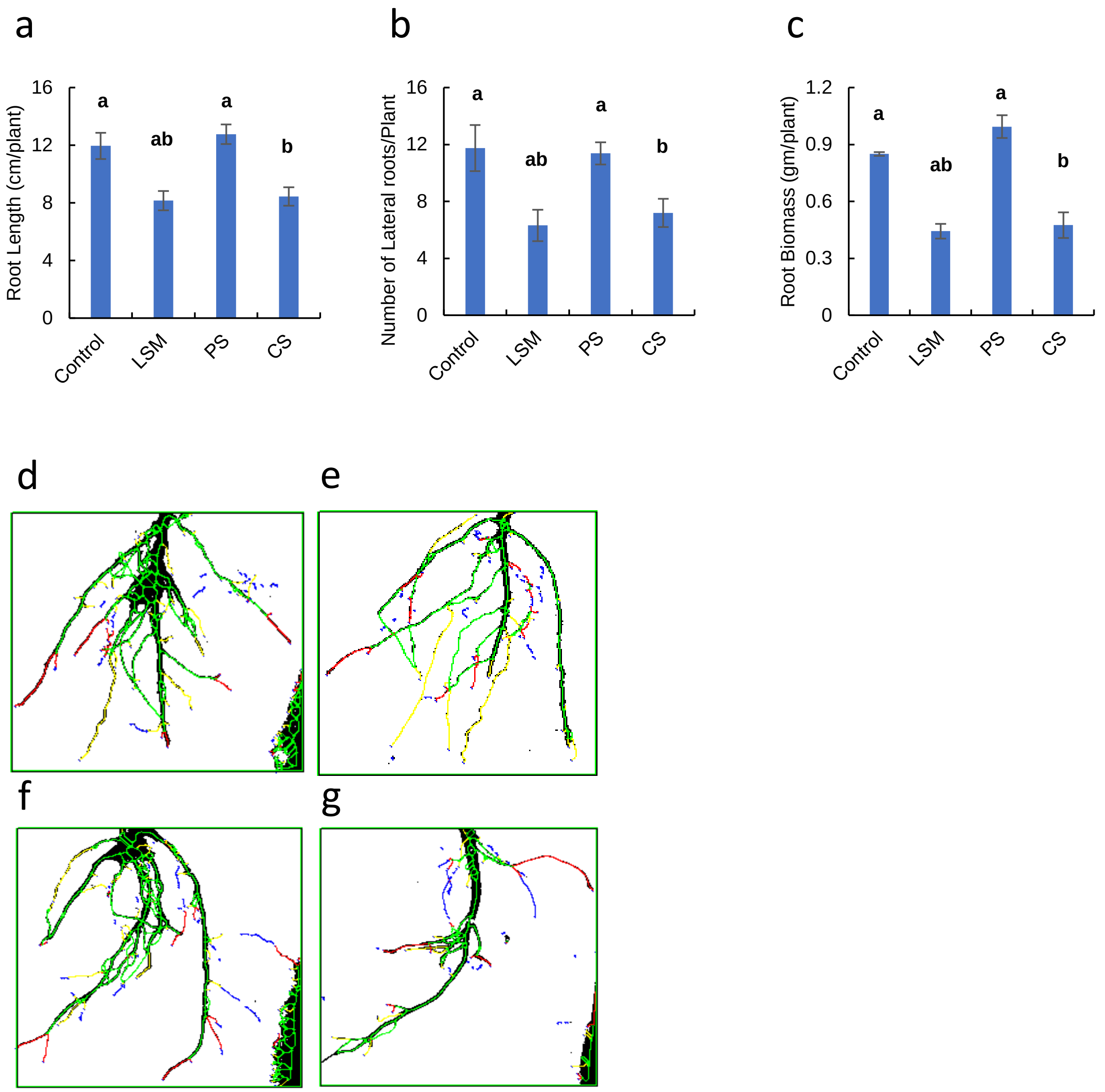

Supplementary Figure S11. Root morphology/phenotype/biomass under different treatments in different year/location field trials. Root length (a), number of lateral root (b) and root biomass (c) was calculated for field trial year 2019-20 for Locations 4. Each bar in graphs is the average of four RCB replicates with SEM as an error bar. Statistical significance difference between means is checked by one way ANOVA and Tukey's Posthoc test. The different letters denotes a significant difference between mean at $p<0.05$. Root architecture was analysed by root system imaging using WinRHIZO professional software for details of root morphology and two dimensional architectural parameters, total root length(TRL), root surface area(RSA), root volume (RV), root diameter(RD), number tips (NT), number of forks(NF), number of cross (NC) and number of links (NL) for all the four treatments (d-h). Total root length distribution is precisely categorized into different diameter class and different colours are used to draw the root skeletons with different diameter class (d-g). Roots overlap at forks and tips are taken into account to provide accurate measurement of total root length and area (h). All the root architectural parameters is average of three to four RCB replicates with standard error of mean. LSM, low soil moisture; PS, Pathogen stress; CS, combined stress

Supplementary Figure S12

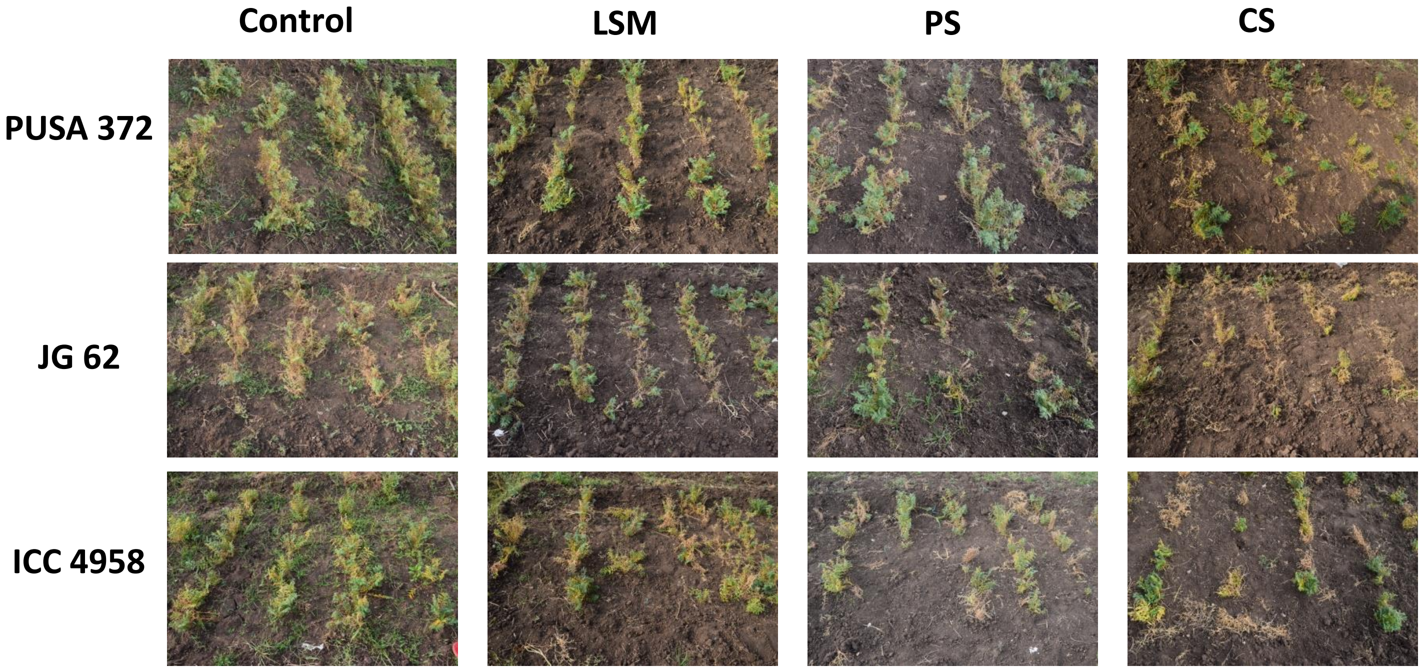

Supplementary Figure S12. DRR disease incidence in various genotypes grown under combined stress in the field. A field study was conducted in Yogi Vemanna University, Kadapa, to assess the effect of soil moisture on dry root rot disease in chickpea cultivation in the year 2019-20. Chickpea was cultivated under rain-fed conditions. PUSA 372, JG 62, and ICC 4958 genotypes were sown in the field, and observations were taken during the harvesting stage. Soil moisture (%) was measured using soil moisture meter Lutron PMS-714. Infected plants were easy to pull out as they are lack of lateral roots and had brittle primary roots. LSM, low soil moisture; PS, Pathogen stress; CS, combined stress

Supplementary figure S13

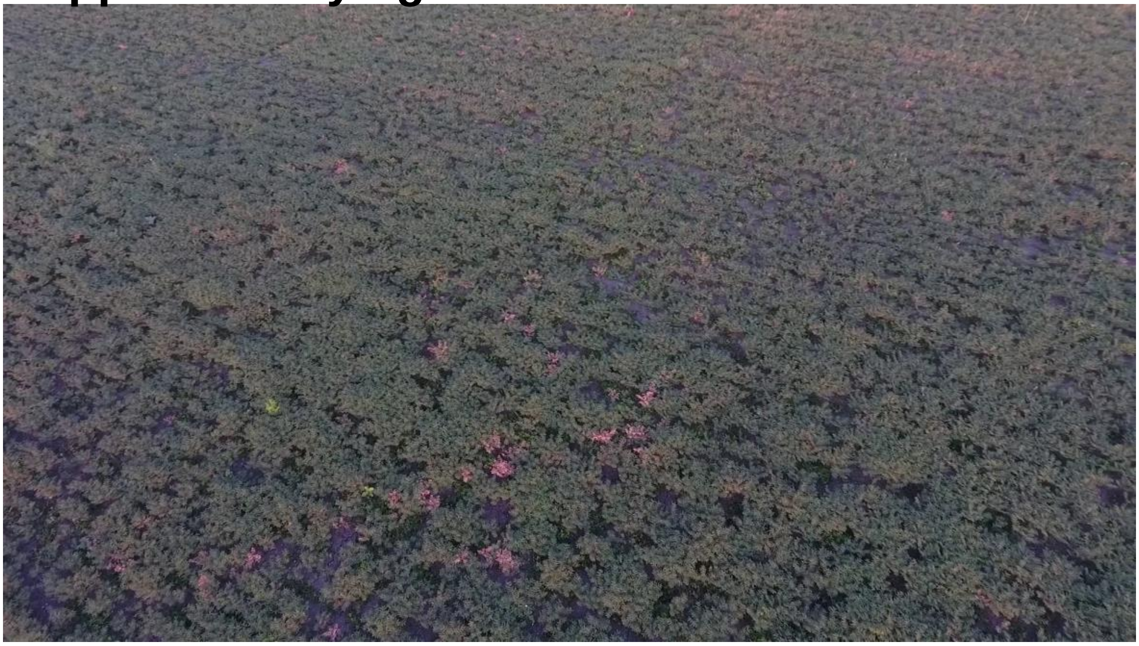

DI of 3%

Sufficient moisture received field

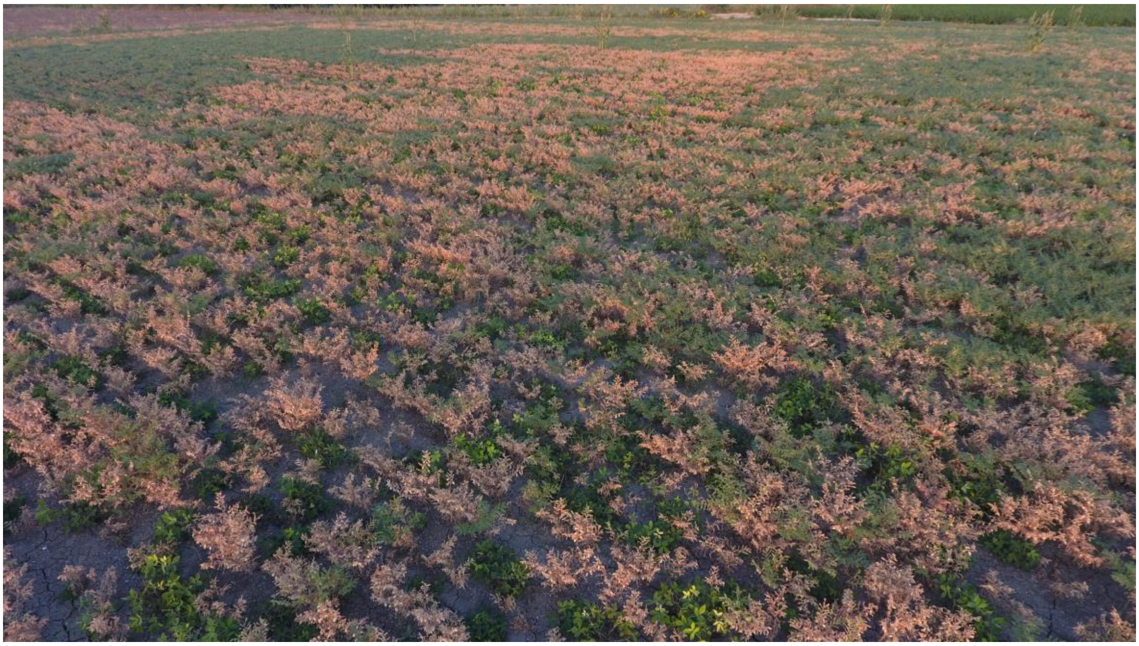

DI of 61.3±3%

Less moisture received field

Supplementary Figure S13. Low soil moisture increases dry root rot in field-grown chickpea. A field survey was conducted in chickpea growing regions across India. An ariel field view with the data from a representative location at Andhra Pradesh is presented here (Anantapur, 14.922662, 77.263522). The data shows the severity of DRR incidence in the year 2019-20. The JG 11 variety was sown in the field and observations were taken during the pod filling stage. Soil moisture (%) was measured using soil moisture meter Lutron PMS-714. Well-irrigated and less-irrigated plots had >40% and <8% soil moisture respectively. Fields with well-irrigation (a) had DRR disease incidence of around 3% and fields which received less irrigation (b) had DRR disease incidence of 61.3±3%. Four random plots with three square meter were selected and disease incidence was calculated (see formula below). Infected plants were easy to pull out as they lacked lateral roots and had brittle primary root. The numbers next to locations are longitude and latitude of the location respectively.

% Disease Incidence= (Number of infected plants/total number of plants)X100.

Acknowledgment: Anantapur (in the farm of Mr. Sudhakar Boya). Details of the field can be found in this video - https://www.youtube.com/watch?v=zZ4VyD4w-kU&t=2s

Supplementary figure S14

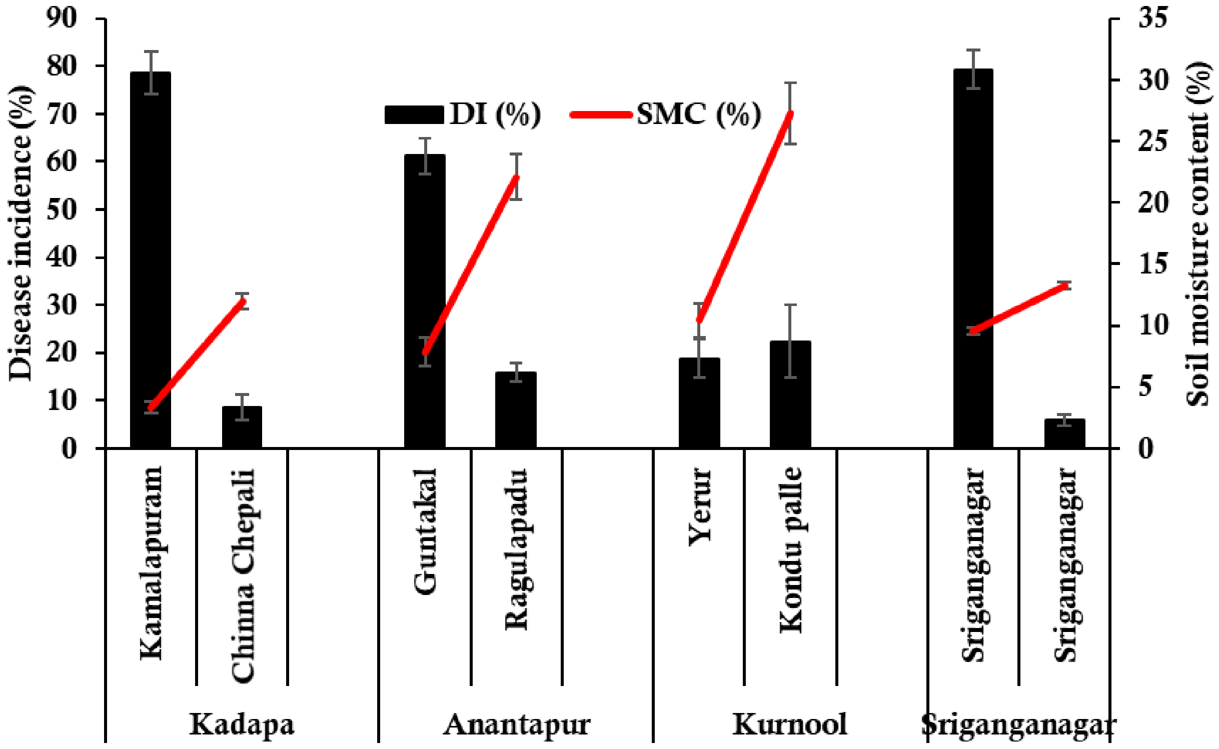

Supplementary Figure S14. Dry root rot incidence observed in representative chickpea fields. A field survey was conducted in chickpea growing regions in Andhra Pradesh (Kadapa (Kamalapuram, 14.545294, 78.593204; china chepali, 14.538757, 78.567917), Anantapur (Guntakal, 15.12121, 77.353083; Ragulapadu, 14.991861, 77.354283), and Kurnool (Yerur, 15.209901, 77.287283; Kondu palle, 15.114348, 78.461722) to assess the effect of soil moisture on dry root rot disease in chickpea cultivation in year 2019-20. Chickpea variety JG 11 was cultivated under rain-fed condition in these locations. Observations were taken during flowering/pod filling stage. Soil moisture (%) was measured using soil moisture meter Lutron PMS-714. Four random plots with three square meter were selected and disease incidence was calculated by method mentioned in **Supplementary Figure S13**. Infected plants were easy to pull out as they are lack of lateral roots and had brittle primary root. Bars in the graph represent average of percent disease incidence for four location within three square meter area and error bar represents SEM. Soil moisture data is average of five different locations within the selected region. The numbers next to locations are longitude and latitude of the location respectively.

Acknowledgment: Anantapur (in the farm of Mr. Jangadigodla Chandra), Kurnool (in the farm of Mr. Lakshmanna Chowdary), Kadappa (in the farm of Mr. Rajasekar Reddy), Sriganganagar (in the field of an agriculture research station, Sriganganagar).

Supplementary Table S1: List of research articles and theses that have data of dry root rot incidence that were used for the simulation study.

S. No.	Authors	Article Title	Journal	Vol & Page No	Location of Field Experiment	Year of Field Trial (Rabi Season; Oct-Mar)
1	Sharma and Kumari[1]	Management of dry root rot disease [*Rhizoctonia bataticola*] of chickpea through fungicides	International Journal of Chemical Studies	5(4): 45-47	Jaipur	2014-15 2015-16
2	Dhingani and Solanky[2]	Integrated Management of Root Rot Disease [*Macrophomina phaseolina* (Tassi.) Goid] of Chickpea through Bioagents, Oil Cakes and Chemicals under Field Conditions in South Gujarat Conditions	Plant Archives	16:183-186	Navsari Agricultural University	2010-11
3	Nagamani et al.[3]	Management of Dry Root Rot Caused By *Rhizoctonia bataticola* (Taub.) Butler in Chickpea	Current Biotica	5(3): 364-369	Tadipatri, Anantapur Andhara Pradesh, India	2009-2011
4	Deepa et al.[4]	Integrated Management of Dry Root Rot Caused by	International Journal of Current Microbiology and Applied Sciences	7(4): 201-209	UAS, Raichur	2015-16

		Rhizoctonia bataticola in Chickpea				
5	Deepa et al.[5]	Distribution and Severity of Dry Root Rot of Chickpea Caused by *Rhizoctonia bataticola* in Parts of North Karnataka, India	International Journal of Current Microbiology and Applied Sciences	7(4): 194-200	Eight chickpea growing districts in North Karnataka	2015-16
6	Khan et al.[6]	Management of Chickpea (*Cicer arietinum* L.) Dry root Rot Caused by *Rhizoctonia bataticola* (Taub.) Butler.	International Journal of Research in Pharmaceutica l and Biomedical Sciences	3 (4); 1539-1548	Various villages in Jammu and Kashmir	2010-11
7	Pandey et al.[7]	Management of Wilt and Root Rot of Chickpea caused by *Fusarium oxysporum* f. sp. *ciceri* and *Macrophomin a phaseolina* through Seed Biopriming and Soil Application of Bio-Agents	International Journal of Current Microbiology and Applied Sciences	6(5): 2516-2522	Anand Agricultural University, Anand, Gujrat	2013-14 2014-15
8	Tinkulal Muchhala[8]	Studies on dry root rot [*Rhizoctonia bataticola* (Taub.) Butler] of chickpea	A thesis submitted to Rajmata Vijayaraje Scindia Krishi Vishwa Vidyalaya, Gwalior		Indore (M.P.)	2017-18
9	Pushpanj ali Wagh[9]	Studies on Dry Root Rot (*Rhizoctonia bataticola*	Thesis Submitted to Department of		Raipur (C.G.) and various districts in	2012-14

		Taub (Butler)) of Chickpea (*Cicer arietinum*).	Plant Pathology College of Agriculture Indira Gandhi Krishi Vishwavidyalaya Raipur (Chhattisgarh)		Chhattisgarh.	
10	Kadam et al.[10]	Survey of Dry Root Rot of Chickpea Incidence in Marathwada Region	Journal of Pharmacognosy and Phytochemistry	SP1: 3004-3008	Various districts in Marathawada region.	2014-15
11	P. Srinivas[11]	Studies on Dry Root Rot [*Rhizoctonia bataticola* (Taub.) Butler] of Chickpea (*Cicer arietinum* L.)	Thesis Submitted to The Professor JayaShankar Telangana State Agricultural University		23 districts of the five major chickpea growing states in central and southern parts of India viz. Andhra Pradesh, Karnataka, Maharashtra, Madhya Pradesh, and Telangana	2013-14 2014-15

1. Sharma, O. P. Management of dry root rot disease [Rhizoctonia bataticola] of chickpea through fungicides. *Int. J. Chem. Stud.* **5**, 45–47 (2017).

2. Dhingani, J. C. & Solanky, K. U. Integrated management of root rot disease [Macrophomina Phaseolina (Tassi.) Goid] of chickpea through bioagents, oil cakes and chemicals under field conditions in South Gujarat Conditions. *Plant Arch.* (2016).

3. Nagamani, P., Viswanath, K. & Babu, T. K. Management of dry root rot caused by Rhizoctonia bataticola (Taub.) Butler in chickpea. *Curr. Biot.* (2011).

4. pa, D. *et al.* Integrated Management of Dry Root Rot Caused by Rhizoctonia bataticola in Chickpea. *Int. J. Curr. Microbiol. Appl. Sci.* (2018). doi:10.20546/ijcmas.2018.704.022

5. pa, D. *et al.* Distribution and Severity of Dry Root Rot of Chickpea Caused by Rhizoctonia bataticola in Parts of North Karnataka. *Int. J. Curr. Microbiol. Appl. Sci.* (2018). doi:10.20546/ijcmas.2018.704.021

6. Khan, R. A., Bhat, T. A. & Kumar, K. Management of Chickpea (Cicer arietinum L .) Dry root Rot Caused by Rhizoctonia bataticola (Taub .) Butler . **3**, 1539–1548 (2012).

7. Pandey, R. N., Gohel, N. M. & Jaisani, P. Management of Wilt and Root Rot of Chickpea caused by Fusarium oxysporum f. sp. ciceri and Macrophomina phaseolina through Seed Biopriming and Soil Application of Bio-Agents. *Int. J. Curr. Microbiol. Appl. Sci.* (2017). doi:10.20546/ijcmas.2017.605.282

8. Muchhala, T. Studies on dry root rot [Rhizoctonia bataticola (Taub.) Butler] of chickpea. (2018).

9. Wagh, P. Studies on dry root rot (Rhizoctonia bataticola Taub (Butler)) of chickpea (Cicer arietinum). (2015).

10. Kadam, A., Chavan Sau, S., Dhutraj Sau, D. & Rewale Sau, K. Survey of dry root rot of chickpea incidence in Marathwada region. ~ *3004 ~ J. Pharmacogn. Phytochem.* 1 (2018).

11. Srinivas, P. Studies on dry root rot [Rhizoctonia bataticola (Taub.) Butler] of chickpea (Cicer arietinum L.). (2016).

Supplementary Table S2a: combinations of input and output parameters used for ANN training and evaluation of neural network prediction accuracy with test set*

Output	Input 1	Input 2	Input 3	Input 4	RMSE	r	R^2
DRR_DI	Variety	Soil_type	RainF_Oct		10.388	0.462	0.214
DRR_DI	Variety	Soil_type	RainF_Nov		8.586	0.655	0.428
DRR_DI	Variety	Soil_type	RainF_Dec		10.077	0.509	0.259
DRR_DI	Variety	Soil_type	RainF_Jan		8.228	0.685	0.469
DRR_DI	Variety	Soil_type	RainF_Feb		8.925	0.587	0.345
DRR_DI	Variety	Soil_type	RainF_OctNov		8.665	0.659	0.434
DRR_DI	Variety	Soil_type	RainF_NovDec		8.330	0.659	0.434
DRR_DI	Variety	Soil_type	RainF_DecJan		8.482	0.670	0.449
DRR_DI	Variety	Soil_type	RainF_JanFeb		8.760	0.637	0.406
DRR_DI	Variety	Soil_type	RainF_OctNovDec		9.406	0.583	0.339
DRR_DI	Variety	Soil_type	RainF_NovDecJan		7.705	0.727	0.528
DRR_DI	Variety	Soil_type	RainF_DecJanFeb		10.241	0.430	0.185
DRR_DI	Variety	Soil_type	MinT_Oct		11.015	0.428	0.183
DRR_DI	Variety	Soil_type	MinT_Nov		9.463	0.550	0.302
DRR_DI	Variety	Soil_type	MinT_Dec		8.418	0.650	0.423
DRR_DI	Variety	Soil_type	MinT_Jan		10.100	0.477	0.228
DRR_DI	Variety	Soil_type	MinT_Feb		10.460	0.447	0.200
DRR_DI	Variety	Soil_type	MinT_OctNov		11.689	0.353	0.124
DRR_DI	Variety	Soil_type	MinT_NovDec		9.328	0.535	0.287
DRR_DI	Variety	Soil_type	MinT_DecJan		9.254	0.573	0.329
DRR_DI	Variety	Soil_type	MinT_JanFeb		10.031	0.533	0.284
DRR_DI	Variety	Soil_type	MinT_OctNovDec		9.034	0.571	0.326
DRR_DI	Variety	Soil_type	MinT_NovDecJan		9.302	0.542	0.294

DRR_DI	Variety	Soil_type	MinT_DecJanFeb		9.846	0.480	0.230
DRR_DI	Variety	Soil_type	MaxT_Oct		8.255	0.663	0.440
DRR_DI	Variety	Soil_type	MaxT_Nov		8.897	0.607	0.368
DRR_DI	Variety	Soil_type	MaxT_Dec		8.630	0.634	0.401
DRR_DI	Variety	Soil_type	MaxT_Jan		10.667	0.458	0.210
DRR_DI	Variety	Soil_type	MaxT_Feb		7.747	0.709	0.503
DRR_DI	Variety	Soil_type	MaxT_OctNov		9.207	0.572	0.327
DRR_DI	Variety	Soil_type	MaxT_NovDec		9.689	0.497	0.247
DRR_DI	Variety	Soil_type	MaxT_DecJan		9.238	0.572	0.327
DRR_DI	Variety	Soil_type	MaxT_JanFeb		10.784	0.461	0.212
DRR_DI	Variety	Soil_type	MaxT_OctNovDec		10.392	0.540	0.292
DRR_DI	Variety	Soil_type	MaxT_NovDecJan		9.333	0.539	0.291
DRR_DI	Variety	Soil_type	MaxT_DecJanFeb		9.689	0.527	0.278
DRR_DI	Variety	Soil_type	RainF_Oct	MaxT_Oct	6.613	0.837	0.701
DRR_DI	Variety	Soil_type	RainF_Oct	MaxT_Nov	9.091	0.633	0.400
DRR_DI	Variety	Soil_type	RainF_Oct	MaxT_Dec	9.472	0.549	0.301
DRR_DI	Variety	Soil_type	RainF_Oct	MaxT_Jan	9.397	0.560	0.313
DRR_DI	Variety	Soil_type	RainF_Oct	MaxT_Feb	9.124	0.613	0.376
DRR_DI	Variety	Soil_type	RainF_Oct	MaxT_OctNov	9.081	0.630	0.397
DRR_DI	Variety	Soil_type	RainF_Oct	MaxT_NovDec	9.414	0.561	0.315
DRR_DI	Variety	Soil_type	RainF_Oct	MaxT_DecJan	9.789	0.547	0.300
DRR_DI	Variety	Soil_type	RainF_Oct	MaxT_JanFeb	11.193	0.447	0.200
DRR_DI	Variety	Soil_type	RainF_Oct	MaxT_OctNovDec	8.573	0.644	0.415
DRR_DI	Variety	Soil_type	RainF_Oct	MaxT_NovDecJan	9.088	0.593	0.352
DRR_DI	Variety	Soil_type	RainF_Oct	MaxT_DecJanFeb	9.467	0.555	0.308
DRR_DI	Variety	Soil_type	RainF_Nov	MaxT_Oct	12.148	0.422	0.178
DRR_DI	Variety	Soil_type	RainF_Nov	MaxT_Nov	22.923	0.084	0.007

DRR_DI	Variety	Soil_type	RainF_Nov	MaxT_Dec	8.350	0.684	0.468
DRR_DI	Variety	Soil_type	RainF_Nov	MaxT_Jan	11.338	0.471	0.222
DRR_DI	Variety	Soil_type	RainF_Nov	MaxT_Feb	9.748	0.556	0.309
DRR_DI	Variety	Soil_type	RainF_Nov	MaxT_OctNov	14.993	0.298	0.089
DRR_DI	Variety	Soil_type	RainF_Nov	MaxT_NovDec	7.075	0.777	0.603
DRR_DI	Variety	Soil_type	RainF_Nov	MaxT_DecJan	8.438	0.676	0.457
DRR_DI	Variety	Soil_type	RainF_Nov	MaxT_JanFeb	10.200	0.546	0.298
DRR_DI	Variety	Soil_type	RainF_Nov	MaxT_OctNovDec	12.898	0.402	0.162
DRR_DI	Variety	Soil_type	RainF_Nov	MaxT_NovDecJan	13.396	0.393	0.154
DRR_DI	Variety	Soil_type	RainF_Nov	MaxT_DecJanFeb	11.928	0.431	0.186
DRR_DI	Variety	Soil_type	RainF_Dec	MaxT_Oct	8.557	0.666	0.443
DRR_DI	Variety	Soil_type	RainF_Dec	MaxT_Nov	11.004	0.430	0.185
DRR_DI	Variety	Soil_type	RainF_Dec	MaxT_Dec	8.876	0.634	0.403
DRR_DI	Variety	Soil_type	RainF_Dec	MaxT_Jan	9.377	0.567	0.322
DRR_DI	Variety	Soil_type	RainF_Dec	MaxT_Feb	9.676	0.524	0.275
DRR_DI	Variety	Soil_type	RainF_Dec	MaxT_OctNov	25.320	0.040	0.002
DRR_DI	Variety	Soil_type	RainF_Dec	MaxT_NovDec	9.641	0.537	0.289
DRR_DI	Variety	Soil_type	RainF_Dec	MaxT_DecJan	9.593	0.551	0.304
DRR_DI	Variety	Soil_type	RainF_Dec	MaxT_JanFeb	9.971	0.513	0.263
DRR_DI	Variety	Soil_type	RainF_Dec	MaxT_OctNovDec	9.892	0.532	0.283
DRR_DI	Variety	Soil_type	RainF_Dec	MaxT_NovDecJan	9.205	0.580	0.336
DRR_DI	Variety	Soil_type	RainF_Dec	MaxT_DecJanFeb	10.241	0.496	0.246
DRR_DI	Variety	Soil_type	RainF_Jan	MaxT_Oct	6.776	0.791	0.625
DRR_DI	Variety	Soil_type	RainF_Jan	MaxT_Nov	7.680	0.727	0.528
DRR_DI	Variety	Soil_type	RainF_Jan	MaxT_Dec	19.564	0.113	0.013
DRR_DI	Variety	Soil_type	RainF_Jan	MaxT_Jan	8.151	0.684	0.468
DRR_DI	Variety	Soil_type	RainF_Jan	MaxT_Feb	7.848	0.705	0.497

DRR_DI	Variety	Soil_type	RainF_Jan	MaxT_OctNov	7.063	0.772	0.597
DRR_DI	Variety	Soil_type	RainF_Jan	MaxT_NovDec	49.912	-0.181	0.033
DRR_DI	Variety	Soil_type	RainF_Jan	MaxT_DecJan	14.313	0.302	0.091
DRR_DI	Variety	Soil_type	RainF_Jan	MaxT_JanFeb	8.220	0.672	0.451
DRR_DI	Variety	Soil_type	RainF_Jan	MaxT_OctNovDec	7.171	0.759	0.576
DRR_DI	Variety	Soil_type	RainF_Jan	MaxT_NovDecJan	14.793	0.251	0.063
DRR_DI	Variety	Soil_type	RainF_Jan	MaxT_DecJanFeb	8.587	0.644	0.415
DRR_DI	Variety	Soil_type	RainF_Feb	MaxT_Oct	8.503	0.665	0.442
DRR_DI	Variety	Soil_type	RainF_Feb	MaxT_Nov	7.373	0.739	0.547
DRR_DI	Variety	Soil_type	RainF_Feb	MaxT_Dec	9.631	0.596	0.355
DRR_DI	Variety	Soil_type	RainF_Feb	MaxT_Jan	10.343	0.507	0.257
DRR_DI	Variety	Soil_type	RainF_Feb	MaxT_Feb	9.546	0.548	0.300
DRR_DI	Variety	Soil_type	RainF_Feb	MaxT_OctNov	8.110	0.676	0.457
DRR_DI	Variety	Soil_type	RainF_Feb	MaxT_NovDec	7.877	0.697	0.486
DRR_DI	Variety	Soil_type	RainF_Feb	MaxT_DecJan	9.462	0.562	0.315
DRR_DI	Variety	Soil_type	RainF_Feb	MaxT_JanFeb	9.570	0.543	0.295
DRR_DI	Variety	Soil_type	RainF_Feb	MaxT_OctNovDec	9.382	0.584	0.341
DRR_DI	Variety	Soil_type	RainF_Feb	MaxT_NovDecJan	8.401	0.649	0.422
DRR_DI	Variety	Soil_type	RainF_Feb	MaxT_DecJanFeb	10.199	0.490	0.240
DRR_DI	Variety	Soil_type	RainF_OctNov	MaxT_Oct	9.665	0.567	0.322
DRR_DI	Variety	Soil_type	RainF_OctNov	MaxT_Nov	10.022	0.557	0.310
DRR_DI	Variety	Soil_type	RainF_OctNov	MaxT_Dec	12.383	0.514	0.264
DRR_DI	Variety	Soil_type	RainF_OctNov	MaxT_Jan	9.069	0.588	0.345
DRR_DI	Variety	Soil_type	RainF_OctNov	MaxT_Feb	9.901	0.552	0.305
DRR_DI	Variety	Soil_type	RainF_OctNov	MaxT_OctNov	9.577	0.572	0.328
DRR_DI	Variety	Soil_type	RainF_OctNov	MaxT_NovDec	10.929	0.451	0.203

DRR_DI	Variety	Soil_type	RainF_OctNov	MaxT_DecJan	8.599	0.631	0.398
DRR_DI	Variety	Soil_type	RainF_OctNov	MaxT_JanFeb	10.560	0.499	0.249
DRR_DI	Variety	Soil_type	RainF_OctNov	MaxT_OctNovDec	10.473	0.426	0.182
DRR_DI	Variety	Soil_type	RainF_OctNov	MaxT_NovDecJan	9.755	0.540	0.291
DRR_DI	Variety	Soil_type	RainF_OctNov	MaxT_DecJanFeb	10.134	0.494	0.244
DRR_DI	Variety	Soil_type	RainF_NovDec	MaxT_Oct	8.780	0.652	0.425
DRR_DI	Variety	Soil_type	RainF_NovDec	MaxT_Nov	8.800	0.643	0.413
DRR_DI	Variety	Soil_type	RainF_NovDec	MaxT_Dec	9.377	0.601	0.361
DRR_DI	Variety	Soil_type	RainF_NovDec	MaxT_Jan	9.947	0.585	0.343
DRR_DI	Variety	Soil_type	RainF_NovDec	MaxT_Feb	9.580	0.549	0.301
DRR_DI	Variety	Soil_type	RainF_NovDec	MaxT_OctNov	8.462	0.659	0.434
DRR_DI	Variety	Soil_type	RainF_NovDec	MaxT_NovDec	8.999	0.654	0.428
DRR_DI	Variety	Soil_type	RainF_NovDec	MaxT_DecJan	9.651	0.634	0.401
DRR_DI	Variety	Soil_type	RainF_NovDec	MaxT_JanFeb	10.157	0.539	0.290
DRR_DI	Variety	Soil_type	RainF_NovDec	MaxT_OctNovDec	8.712	0.658	0.433
DRR_DI	Variety	Soil_type	RainF_NovDec	MaxT_NovDecJan	9.832	0.598	0.357
DRR_DI	Variety	Soil_type	RainF_NovDec	MaxT_DecJanFeb	9.997	0.576	0.332
DRR_DI	Variety	Soil_type	RainF_DecJan	MaxT_Oct	6.769	0.791	0.625
DRR_DI	Variety	Soil_type	RainF_DecJan	MaxT_Nov	10.854	0.452	0.205
DRR_DI	Variety	Soil_type	RainF_DecJan	MaxT_Dec	15.059	0.254	0.065
DRR_DI	Variety	Soil_type	RainF_DecJan	MaxT_Jan	8.382	0.664	0.441
DRR_DI	Variety	Soil_type	RainF_DecJan	MaxT_Feb	7.927	0.700	0.490
DRR_DI	Variety	Soil_type	RainF_DecJan	MaxT_OctNov	7.126	0.767	0.589
DRR_DI	Variety	Soil_type	RainF_DecJan	MaxT_NovDec	11.685	0.406	0.165
DRR_DI	Variety	Soil_type	RainF_DecJan	MaxT_DecJan	8.876	0.639	0.409
DRR_DI	Variety	Soil_type	RainF_DecJan	MaxT_JanFeb	10.145	0.495	0.245
DRR_DI	Variety	Soil_type	RainF_DecJan	MaxT_OctNovDec	8.208	0.680	0.462

DRR_DI	Variety	Soil_type	RainF_DecJan	MaxT_NovDecJan	11.576	0.413	0.171
DRR_DI	Variety	Soil_type	RainF_DecJan	MaxT_DecJanFeb	8.046	0.692	0.479
DRR_DI	Variety	Soil_type	RainF_JanFeb	MaxT_Oct	11.976	0.389	0.151
DRR_DI	Variety	Soil_type	RainF_JanFeb	MaxT_Nov	11.460	0.398	0.158
DRR_DI	Variety	Soil_type	RainF_JanFeb	MaxT_Dec	14.274	0.287	0.082
DRR_DI	Variety	Soil_type	RainF_JanFeb	MaxT_Jan	8.280	0.679	0.461
DRR_DI	Variety	Soil_type	RainF_JanFeb	MaxT_Feb	8.769	0.639	0.408
DRR_DI	Variety	Soil_type	RainF_JanFeb	MaxT_OctNov	6.978	0.776	0.602
DRR_DI	Variety	Soil_type	RainF_JanFeb	MaxT_NovDec	15.951	0.198	0.039
DRR_DI	Variety	Soil_type	RainF_JanFeb	MaxT_DecJan	7.954	0.702	0.493
DRR_DI	Variety	Soil_type	RainF_JanFeb	MaxT_JanFeb	8.298	0.669	0.447
DRR_DI	Variety	Soil_type	RainF_JanFeb	MaxT_OctNovDec	7.888	0.721	0.520
DRR_DI	Variety	Soil_type	RainF_JanFeb	MaxT_NovDecJan	9.014	0.613	0.376
DRR_DI	Variety	Soil_type	RainF_JanFeb	MaxT_DecJanFeb	8.353	0.670	0.448
DRR_DI	Variety	Soil_type	RainF_OctNovDec	MaxT_Oct	9.048	0.606	0.367
DRR_DI	Variety	Soil_type	RainF_OctNovDec	MaxT_Nov	9.093	0.566	0.320
DRR_DI	Variety	Soil_type	RainF_OctNovDec	MaxT_Dec	10.118	0.567	0.321
DRR_DI	Variety	Soil_type	RainF_OctNovDec	MaxT_Jan	9.557	0.546	0.298
DRR_DI	Variety	Soil_type	RainF_OctNovDec	MaxT_Feb	10.058	0.514	0.264
DRR_DI	Variety	Soil_type	RainF_OctNovDec	MaxT_OctNov	8.490	0.631	0.399
DRR_DI	Variety	Soil_type	RainF_OctNovDec	MaxT_NovDec	13.199	0.563	0.317
DRR_DI	Variety	Soil_type	RainF_OctNovDec	MaxT_DecJan	8.689	0.619	0.383
DRR_DI	Variety	Soil_type	RainF_OctNovDec	MaxT_JanFeb	10.764	0.481	0.231
DRR_DI	Variety	Soil_type	RainF_OctNovDec	MaxT_OctNovDec	9.666	0.504	0.254
DRR_DI	Variety	Soil_type	RainF_OctNovDec	MaxT_NovDecJan	10.000	0.520	0.270
DRR_DI	Variety	Soil_type	RainF_OctNovDec	MaxT_DecJanFeb	9.569	0.528	0.278
DRR_DI	Variety	Soil_type	RainF_NovDecJan	MaxT_Oct	12.633	0.347	0.121

DRR_DI	Variety	Soil_type	RainF_NovDecJan	MaxT_Nov	7.040	0.766	0.587
DRR_DI	Variety	Soil_type	RainF_NovDecJan	MaxT_Dec	7.920	0.736	0.541
DRR_DI	Variety	Soil_type	RainF_NovDecJan	MaxT_Jan	7.476	0.745	0.554
DRR_DI	Variety	Soil_type	RainF_NovDecJan	MaxT_Feb	7.700	0.722	0.521
DRR_DI	Variety	Soil_type	RainF_NovDecJan	MaxT_OctNov	7.228	0.766	0.586
DRR_DI	Variety	Soil_type	RainF_NovDecJan	MaxT_NovDec	7.215	0.759	0.576
DRR_DI	Variety	Soil_type	RainF_NovDecJan	MaxT_DecJan	7.754	0.729	0.531
DRR_DI	Variety	Soil_type	RainF_NovDecJan	MaxT_JanFeb	7.643	0.726	0.528
DRR_DI	Variety	Soil_type	RainF_NovDecJan	MaxT_OctNovDec	7.313	0.750	0.562
DRR_DI	Variety	Soil_type	RainF_NovDecJan	MaxT_NovDecJan	7.806	0.728	0.529
DRR_DI	Variety	Soil_type	RainF_NovDecJan	MaxT_DecJanFeb	7.844	0.716	0.512
DRR_DI	Variety	Soil_type	RainF_DecJanFeb	MaxT_Oct	6.883	0.787	0.619
DRR_DI	Variety	Soil_type	RainF_DecJanFeb	MaxT_Nov	9.481	0.550	0.303
DRR_DI	Variety	Soil_type	RainF_DecJanFeb	MaxT_Dec	9.431	0.619	0.383
DRR_DI	Variety	Soil_type	RainF_DecJanFeb	MaxT_Jan	8.324	0.676	0.457
DRR_DI	Variety	Soil_type	RainF_DecJanFeb	MaxT_Feb	8.732	0.628	0.395
DRR_DI	Variety	Soil_type	RainF_DecJanFeb	MaxT_OctNov	7.185	0.760	0.578
DRR_DI	Variety	Soil_type	RainF_DecJanFeb	MaxT_NovDec	7.453	0.738	0.545
DRR_DI	Variety	Soil_type	RainF_DecJanFeb	MaxT_DecJan	8.121	0.692	0.478
DRR_DI	Variety	Soil_type	RainF_DecJanFeb	MaxT_JanFeb	8.403	0.667	0.445
DRR_DI	Variety	Soil_type	RainF_DecJanFeb	MaxT_OctNovDec	7.855	0.708	0.501
DRR_DI	Variety	Soil_type	RainF_DecJanFeb	MaxT_NovDecJan	8.347	0.659	0.434
DRR_DI	Variety	Soil_type	RainF_DecJanFeb	MaxT_DecJanFeb	7.721	0.717	0.513

*ANN was trained with combination of three or four input parameters and DRR disease incidence as output. Their prediction accuracy was checked with test sets by calculating root mean squared error (RMSE), correlation coefficient (r) and coefficient of determination (R^2).

Supplementary Table S2b: K-fold cross validation of neural network trained with selected combination of input and output variables to select best fit neural network*

Output	Inputs				10-fold Correlation coefficient			
Output	Input-1	Input-2	Input-3	Input-4	Average of 10-fold	Maximum of 10-fold	Minimum of 10-fold	Range of 10-fold
DRR_DI	Variety	Soil_Number	RainF_NovDecJan	MaxT_Oct	0.768	0.875	0.609	0.074
DRR_DI	Variety	Soil_Number	RainF_NovDecJan	MaxT_Nov	0.751	0.889	0.662	0.069
DRR_DI	Variety	Soil_Number	RainF_NovDecJan	MaxT_Dec	0.672	0.855	0.603	0.071
DRR_DI	Variety	Soil_Number	RainF_NovDecJan	MaxT_Jan	0.632	0.805	0.378	0.122
DRR_DI	Variety	Soil_Number	RainF_NovDecJan	MaxT_Feb	0.721	0.871	0.431	0.126
DRR_DI	Variety	Soil_Number	RainF_NovDecJan	MaxT_OctNov	0.772	0.903	0.580	0.087
DRR_DI	Variety	Soil_Number	RainF_NovDecJan	MaxT_NovDec	0.720	0.873	0.607	0.082
DRR_DI	Variety	Soil_Number	RainF_NovDecJan	MaxT_DecJan	0.678	0.828	0.599	0.069
DRR_DI	Variety	Soil_Number	RainF_NovDecJan	MaxT_JanFeb	0.667	0.822	0.395	0.134
DRR_DI	Variety	Soil_Number	RainF_NovDecJan	MaxT_OctNovDec	0.687	0.814	0.370	0.136
DRR_DI	Variety	Soil_Number	RainF_NovDecJan	MaxT_NovDecJan	0.702	0.858	0.393	0.127
DRR_DI	Variety	Soil_Number	RainF_NovDecJan	MaxT_DecJanFeb	0.666	0.848	0.450	0.115
DRR_DI	Variety	Soil_Number	RainF_DecJanFeb	MaxT_Oct	0.756	0.875	0.669	0.065
DRR_DI	Variety	Soil_Number	RainF_DecJanFeb	MaxT_Nov	0.732	0.857	0.624	0.083
DRR_DI	Variety	Soil_Number	RainF_DecJanFeb	MaxT_Dec	0.568	0.786	0.440	0.110
DRR_DI	Variety	Soil_Number	RainF_DecJanFeb	MaxT_Jan	0.595	0.784	0.468	0.095
DRR_DI	Variety	Soil_Number	RainF_DecJanFeb	MaxT_Feb	0.664	0.845	0.558	0.120
DRR_DI	Variety	Soil_Number	RainF_DecJanFeb	MaxT_OctNov	0.769	0.871	0.690	0.071

DRR_DI	Variety	Soil_Number	RainF_DecJanFeb	MaxT_NovDec	0.666	0.831	0.535	0.098
DRR_DI	Variety	Soil_Number	RainF_DecJanFeb	MaxT_DecJan	0.625	0.793	0.492	0.099
DRR_DI	Variety	Soil_Number	RainF_DecJanFeb	MaxT_JanFeb	0.675	0.824	0.498	0.125
DRR_DI	Variety	Soil_Number	RainF_DecJanFeb	MaxT_OctNovDec	0.728	0.841	0.596	0.087
DRR_DI	Variety	Soil_Number	RainF_DecJanFeb	MaxT_NovDecJan	0.675	0.824	0.538	0.104
DRR_DI	Variety	Soil_Number	RainF_DecJanFeb	MaxT_DecJanFeb	0.660	0.858	0.468	0.122

* k-fold cross validation (k=10) carried out to select best fit models out of 24 neural networks trained with combination of input as shown above. We represent here average correlation coefficient of 10-fold neural networks for each input combinations along with maximum, minimum and range of correlation coefficient of 10-fold neural networks.

Supplementary Table S3a: Soil characteristics and DRR disease incidence from different field trial of year 2019-20

Locations	Nitrogen	Phosphorus	Potassium	Organic Carbon	Electrical Conductivity	%Sand	%Silt	%Clay	DRR-DI
**Location-1**	119.00	28.60	230.00	0.22	0.14	20.00	54.00	26.00	31.25
	119.00	28.60	230.00	0.22	0.14	20.00	54.00	26.00	26.92
	119.00	28.60	230.00	0.22	0.14	20.00	54.00	26.00	34.29
**Location-4**	170.00	50.90	348.00	0.63	0.24	30.00	42.00	28.00	50.00
	170.00	50.90	348.00	0.63	0.24	30.00	42.00	28.00	48.03
	170.00	50.90	348.00	0.63	0.24	30.00	42.00	28.00	50.85
	170.00	50.90	348.00	0.63	0.24	30.00	42.00	28.00	48.95
**Location-3**	224.00	35.80	124.00	0.38	1.06	50.00	30.00	20.00	29.82
	224.00	35.80	124.00	0.38	1.06	50.00	30.00	20.00	43.42
	224.00	35.80	124.00	0.38	1.06	50.00	30.00	20.00	38.46
**Location-2**	176.00	68.40	338.00	0.66	0.29	46.00	15.00	39.00	80.09
	176.00	68.40	338.00	0.66	0.29	46.00	15.00	39.00	81.50
	176.00	68.40	338.00	0.66	0.29	46.00	15.00	39.00	88.27

Supplementary Table S3b: Correlation coefficient between soil factors and DRR disease incidence

	Nitrogen	Phosphorus	Potassium	Organic C	EC	Sand	Silt	Clay	DRR-DI
**Nitrogen**	1.00	0.21	-0.39	0.32	0.87	0.90	-0.63	-0.26	0.16
**Phosphorus**	0.21	1.00	0.73	0.92	-0.25	0.43	-0.76	0.86	0.96
**Potassium**	-0.39	0.73	1.00	0.75	-0.79	-0.29	-0.12	0.77	0.62
**Organic C**	0.32	0.92	0.75	1.00	-0.18	0.37	-0.61	0.64	0.79
**EC**	0.87	-0.25	-0.79	-0.18	1.00	0.75	-0.35	-0.59	-0.23
**Sand**	0.90	0.43	-0.29	0.37	0.75	1.00	-0.88	0.09	0.46
**Silt**	-0.63	-0.76	-0.12	-0.61	-0.35	-0.88	1.00	-0.55	-0.81
**Clay**	-0.26	0.86	0.77	0.64	-0.59	0.09	-0.55	1.00	0.90

DI	0.16	0.96	0.62	0.79	-0.23	0.46	-0.81	0.90	1.00

Supplementary Table S3c: *p*-value of correlation coefficient

	Nitrogen	Phosphorus	Potassium	Organic C	EC	Sand	Silt	Clay	DRR-DI
**Nitrogen**	NA	0.50110	0.19372	0.28682	0.00010	0.00003	0.02095	0.39035	0.60358
**Phosphorus**	0.50110	NA	0.00463	0.00001	0.40447	0.14603	0.00234	0.00016	0.00000
**Potassium**	0.19372	0.00463	NA	0.00318	0.00144	0.33364	0.69991	0.00203	0.02288
**Organic C**	0.28682	0.00001	0.00318	NA	0.55053	0.21775	0.02661	0.01818	0.00135
**EC**	0.00010	0.40447	0.00144	0.55053	NA	0.00322	0.23818	0.03430	0.45053
**Sand**	0.00003	0.14603	0.33364	0.21775	0.00322	NA	0.00006	0.77213	0.11236
**Silt**	0.02095	0.00234	0.69991	0.02661	0.23818	0.00006	NA	0.05328	0.00078
**Clay**	0.39035	0.00016	0.00203	0.01818	0.03430	0.77213	0.05328	NA	0.00003
**DI**	0.60358	0.00000	0.02288	0.00135	0.45053	0.11236	0.00078	0.00003	NA

Supplementary Table S4: *Rhizoctonia bataticola* inoculum load in soil of field trial locations

**Field locations**	**Susceptible varieties used**	**Disease incidence (%)**	**Category of inoculum load**
Location 1	JG62	40-50	Medium
Location 2	JG62	80-90	High
Location 3	JG62	45-50	Medium
Location 4	BG212	30-35	Medium
Location 5	JG16 and JAK9218	7-10	Low
Location 6	JG62	50-55	High
Location 7	L550 and BG212	30-50	Medium

In order to establish pathogen load in field trial location, chickpea pathogen susceptible genotypes were sown in an area of around 1 m^2. Percent disease incidence were calculated and were categorized in to low pathogen load (5-10%), medium pathogen load (10-50%) and high pathogen load (50-100%).
